# Extraction of Antioxidants from Brown Macroalgae *Fucus spiralis*

**DOI:** 10.3390/molecules29102271

**Published:** 2024-05-11

**Authors:** André Horta, Ana M. Duarte, Sónia Barroso, Filipa R. Pinto, Susana Mendes, Vasco Lima, Jorge A. Saraiva, Maria M. Gil

**Affiliations:** 1 MARE—Marine and Environmental Sciences Centre/ARNET—Aquatic Research Network, School of Tourism and Maritime Technology, Polytechnic of Leiria, Cetemares, 2520-620 Peniche, Portugalfilipa.pinto@ipleiria.pt (F.R.P.);; 2 Division of Aquaculture, Upgrading and Bioprospection, Portuguese Institute for the Sea and Atmosphere (IPMA), Avenida Magalhães Ramalho, 6, 1495-165 Lisbon, Portugal; 3 Research Institute for Medicines, Faculty of Pharmacy, University of Lisbon, Avenida Professor Gama Pinto, 1649-003 Lisbon, Portugal; 4 LAQV-REQUIMTE, Department of Chemistry, University of Aveiro, 3810-193 Aveiro, Portugal

**Keywords:** antioxidants, green extraction, macroalgae, response surface methodology, extraction optimization

## Abstract

In this study, different extraction methods and conditions were used for the extraction of antioxidants from brown macroalgae *Fucus spiralis*. The extraction methodologies used were ultrasound-assisted extraction (ultrasonic bath and ultrasonic probe), extraction with a vortex, extraction with an Ultra-Turrax^®^ homogenizer, and high-pressure-assisted extraction. The extracts were analyzed for their total phenolic content (TPC) and their antioxidant activity, and evaluated through the 2,2-difenil-1-picrilhidrazil (DPPH) free radical scavenging method and ferric reducing antioxidant power (FRAP) assay. Ultrasonic probe-assisted extraction yielded the highest values of TPC (94.78–474.16 mg gallic acid equivalents/g extract). Regarding the antioxidant activity, vortex-assisted extraction gave the best DPPH results (IC_50_ 1.89–16 µg/mL), while the highest FRAP results were obtained using the Ultra-Turrax^®^ homogenizer (502.16–1188.81 μmol ascorbic acid equivalents/g extract). For each extraction method, response surface methodology was used to analyze the influence of the experimental conditions “extraction time” (t), “biomass/solvent ratio” (R), “solvent” (S, water % in water/ethanol mixture), and “pressure” (P) on TPC, DPPH, and FRAP of the *F. spiralis* extracts. In general, higher TPC content and higher antioxidant capacity (lower IC_50_ and higher FRAP) were obtained with higher R, t, and P, and lower S (higher ethanol %). The model regarding the combined effects of independent variables t, R, and S on the FRAP response values for vortex-assisted extractions best fitted the experimental data (*R*^2^ 0.957), with optimal extraction conditions of t = 300 s, R = 50 g, and S = 25%.

## 1. Introduction

The accumulation of harmful free radicals and reactive oxygen species (ROS) in the body can have damaging effects on biologically significant molecules, resulting in the occurrence of several life-threatening diseases [1]. In the past, many antioxidants have been chemically synthesized or purified from natural resources, but natural antioxidants are more desirable as they have been shown to have fewer side effects [2,3]. 

Algae, with their great taxonomic diversity, are a very promising natural source of various bioactive compounds, such as polyphenols, polysaccharides, carotenoids, and polyunsaturated fatty acids [4,5]. Algae are often exposed to extreme environmental conditions of light, salinity, and temperature. In order to adapt, they develop defense mechanisms that produce a variety of important bioactive secondary metabolites, including antioxidant compounds such as phycobiliproteins, phlorotannins, polysaccharides, and carotenoids [6,7]. In recent decades, research into the potential application of algal antioxidants in food, cosmetics, pharmaceuticals, and therapeutic products has revealed very promising results. Bioactive compounds from algae have been reported to have chemoprotective, neuroprotective, anti-inflammatory, antidiabetic, antiproliferative, antibacterial, antiviral, and UV-protective activities [1,8].

The extraction of bioactive compounds from algae is carried out both by conventional methods and by new ecological alternative methods. Conventional cell disruption methods include extraction with water (autoclaving/boiling/homogenization), acid/alkaline hydrolysis, and conventional extraction with solvents (liquid–liquid/solid–liquid/Soxhlet extraction). However, these techniques use large volumes of solvents and have long extraction times, and often produce low extraction yields. Modern extraction techniques include supercritical fluid extraction, ultrasound-assisted extraction, microwave-assisted extraction, nonthermal high pressure extraction, and pressurized liquid extraction [6,9,10]. The use of such advanced ecological extraction techniques can be an effective alternative to the problems encountered with the use of traditional extraction procedures, since they allow for a more efficient extraction of the target compounds, minimizing the use of toxic organic solvents. 

Ultrasound-assisted extraction is based on the application of ultrasonic waves (an ultrasound bath or ultrasound probe) to a matrix immersed in a liquid medium, inducing the rupture of cell walls and the release of the compounds of interest. It is a useful method for the extraction of many biocompounds, generally giving high extraction yields with low solvent and energy consumption [11]. High-pressure-assisted extraction is characterized by the use of high pressure (up to 800 MPa), enabling short processing times and the use of low solvent volumes. This extraction method is considered a green extraction technology and has been used to extract several bioactive compounds from different matrices [12,13]. Although homogenization with a vortex or Ultra-Turrax^®^ is mainly used as a biomass pre-treatment for other extraction techniques, they are themselves potential extraction methods. 

*Fucus spiralis*, an edible brown seaweed found on the Portuguese coast, has been found to have high antioxidant activity, which could be valuable for functional food applications [14,15]. Among other antioxidant compounds, *Fucus spiralis* produces polyphenols such as phlorotannins [16]. In this study, five different methods were used to extract antioxidants from *Fucus spiralis*: ultrasound-assisted extraction (using an ultrasonic bath and an ultrasonic probe), extraction with a vortex, extraction with an Ultra-Turrax^®^ homogenizer, and high-pressure extraction. The influence of experimental conditions, such as solvent, extraction time, biomass/solvent ratio, and pressure, was also analyzed. For each extraction method, response surface methodology (RSM) was used to determine and optimize the optimal experimental conditions to obtain the maximum yield with the minimum extraction time and resource consumption [17]. Food-friendly solvents were used, with a view to future applications of antioxidant-enriched extracts in food matrices.

## 2. Results and Discussion

### 2.1. Extraction Experimental Results

The experimental results obtained using the conditions defined by RSM are shown in Table 1 and Table 2. Analysis of the results obtained for TPC (mg GAE/g extract) showed that the highest values were achieved with ultrasonic probe extraction, with TPC values ranging from 94.78 to 474.16 mg GAE/g of extract (Table 1, runs 8 and 3, respectively), while the worst TPC values were obtained with vortex extraction, with TPC values ranging from 25.04 to 176.26 mg GAE/g of extract (Table 1, runs 10 and 7, respectively). These values agree with those obtained by [18], who reported a TPC of 245 mg GAE/g dry *F. spiralis* collected in July/August on the west coast of Scotland, extracted with 95% ethanol. Other authors have reported much lower TPC values for *F. spiralis* extracted with ethanol or ethanol/water mixtures. Farvin et al. [19] obtained a TPC of 7.52 mg GAE/g dry *F. spiralis* collected between April and September off the coast of Denmark and extracted with 96% ethanol. Bittkau et al. [20] found a TPC content of 25.8 to 35.1 mg GAE/g of ethanolic extracts from *Fucus* samples (*Fucus vesiculosus*, *Fucus serranus*, and *Fucus evanescens*) collected in July in the Baltic Sea. These differences in TPC content may be related to both the harvesting month and location, since several studies have shown that the content of phenolic compounds can vary significantly throughout the seasons and across different geographical locations [21,22]. 

Antioxidant activity was assessed using the DPPH (IC_50_, mg/mL) and FRAP (µmol AAE/g) methods. Regarding the DPPH method, the best results were obtained using vortex-assisted extraction, with IC_50_ values ranging from 1.89 to 16 µg/mL (Table 1, runs 7 and 10, respectively), while the worst results were obtained with high-pressure-assisted extraction, with IC_50_ values ranging from 3.57 to 300 µg/mL (Table 2, runs 20 and 3, respectively). An IC_50_ of 71.5 µg/mL was reported for ethanolic extracts of *F. spiralis* [22], while a higher value, an IC_50_ of 17.71 mg/mL, was reported for ethanol/water extracts of the same species [23].

With regard to the FRAP method, the results obtained for the vortex, ultrasonic bath, ultrasonic probe, and high-pressure extractions were similar, ranging from 96.25 to 518.85 μmol/g of extract (Table 1 and Table 2). The best FRAP results were obtained with Ultra-Turrax^®^ homogenizer extraction, with values ranging from 502.16 to 1188.81 μmol/g of extract (Table 1, runs 6 and 9, respectively).

Dang et al. [24] reported better TPC, DPPH, and FRAP results for ethanolic extracts obtained from the brown algae *Hormosira banksii* using ultrasound-assisted extraction compared to conventional solid–liquid extraction. However, in the present study, the best antioxidant capacity results for FRAP and DPPH were not obtained with the same extraction method, as mentioned above. Although this trend is to be expected, the assays used to measure antioxidant capacity target different molecules, and these molecules may be extracted differently by the different extraction methods.

Regarding vortex-assisted extraction, the best results for the TPC, DPPH, and FRAP assays were obtained in run 7 (Table 1, t = 245 s, R = 40 g biomass/100 mL solvent, S = 20% water in the water/ethanol mixture). Several studies have reported higher TPC content and greater antioxidant capacity in extracts obtained with 80% ethanol, since dual-solvent systems create a polar medium favorable to the extraction of more polyphenols [23]. 

### 2.2. Response Surface Methodology and Statistical Analysis

Quadratic polynomial equations were established on the basis of the experimental results in order to identify the relationship between the independent variables and the response variables. Table 3 shows a summary of the statistically significant effects of the independent variables on the response values (that is, DPPH, FRAP, and TPC) for all the extraction methods used, as well as the mathematical models obtained after fitting the experimental results to Equation (8). Detailed ANOVA, effect estimates, and regression coefficient tables can be found in the Supplementary Material.

#### 2.2.1. Vortex-Assisted Extraction

**Figure 1:**
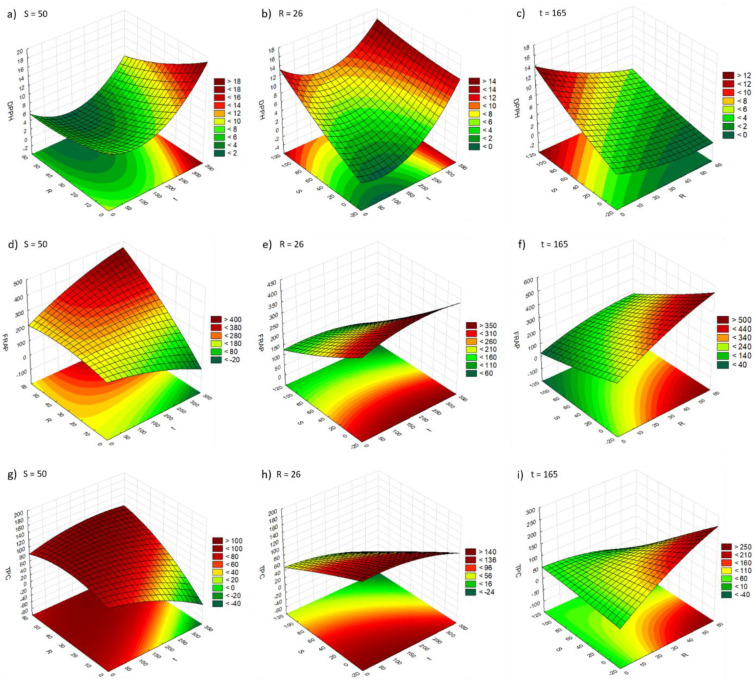
Response surfaces observed for DPPH (**a**–**c**), FRAP (**d**–**f**), and TPC (**g**–**i**) for vortex-assisted extraction. DPPH, FRAP, and TPC results are presented as IC_50_ (µg/mL), µmol AAE/g extract, and mg GAE/g extract, respectively. **t** (s); **R** (g biomass/100 mL solvent); **S** (% water in a water/ethanol mixture).

For vortex-assisted extraction, the model relating the combined effects of the independent variables **t**, **R**, and **S** on the DPPH response values showed a poor fit with the experimental data (*R*^2^ 0.523, *R*^2^ *adj.* 0.000; Table 3 and Table S3). Variables **t** and **S** were found to have a positive linear effect on antioxidant activity using DPPH method, variable **R** had a negative linear effect on DPPH, and no correlation between the independent variables was statistically significant (*p*-value > 0.05, Table 3 and Table S4). When comparing the experimental results with those predicted by the model, large residuals were obtained, showing the poor fit of the model to the experimental data (Figure S2a; Table S6). The results were plotted on response surface graphs for easier visual analysis (Figure 1). In general, IC_50_ values decrease (higher antioxidant capacity) with higher **R**, lower **S**, and medium/low **t** (Figure 1a–c). The regression coefficients of the model regarding the DPPH response to the independent variables were all non-significant (*p* > 0.05; Table S5), and therefore the model equation for Y_DPPH_ is not shown. 

For the FRAP assay, the model relating the combined effects of the independent variables **t**, **R**, and **S** on the FRAP response values (Equation (1); Table 3) showed high significance (statistically significant effects of the independent variables, *p* < 0.05) and a good fit to the experimental data (*R*^2^ 0.957, *R*^2^ *adj.* 0.892; Table 3 and Table S3). Variable **R** had a positive linear effect and a negative quadratic effect on FRAP result, while variable **S** had a negative linear effect and variable **t** had a negative quadratic effect (*p <* 0.05, Table 3 and Table S4). The correlations between variables **t** and **R**, and between variables **R** and **S** were also statistically significant (*p <* 0.05, Table 3 and Table S4). When comparing the experimental results with those predicted by the model, small residuals were obtained, revealing a good fit of the model to the experimental data (Figure S2b; Table S6). Variables **R** and **S** had the greatest influence on the FRAP results. An increase in the % of water in the extraction solvent (**S**) from 0 to 100% led to a 52% decrease in the FRAP result (t = 300 s, R = 50 g/100 mL), while an increase in the biomass/solvent ratio (**R**) from 2 to 50 g/100 mL solvent led to a 78% decrease in the FRAP result. In general, higher **R** and lower **S** resulted in higher FRAP values, while **t** had no significant influence (Figure 1d–f).

Regarding the TPC assay, the model describing the combined effects of the independent variables **t**, **R**, and **S** on the TPC response values showed low significance and a poor fit to the experimental data (*R*^2^ 0.762, *R*^2^ *adj.* 0.405; Table 3 and Table S3). None of the independent variables **t**, **R**, and **S** had a significant effect (*p*-value > 0.05) on the TPC value (Table 3 and Table S4), and therefore no model equation is presented for Y_TPC_. Furthermore, when comparing the experimental results with those predicted by the model, large residuals are obtained, which reveal a poor fit of the model to the experimental data (Figure S2c; Table S6). Looking at the 3D response surfaces, a higher TPC response is obtained with higher values of **R** and lower values of **t** and **S** values (Figure 1g–i). 

#### 2.2.2. Extraction with Ultra-Turrax^®^ Homogenizer

For extractions with the Ultra-Turrax^®^ homogenizer, the models for the combined effects of the independent variables **t**, **R**, and **S** on the FRAP response values showed low significance and a weak fit to the experimental data (*R*^2^ 0.466, *R*^2^ *adj.* 0.000; Table 3 and Table S7). When comparing the experimental results with those predicted by the model, large residuals were obtained, revealing a poor fit of the model to the experimental data (Figure S4b; Table S10). The models for DPPH and TPC showed a good fit with the experimental data (*R*^2^ 0.899, *R*^2^ *adj.* 0.747 for the DPPH model; *R*^2^ 0.857, *R*^2^ *adj.* 0.642 for the TPC model; Table 3 and Table S7), and small residuals were obtained when comparing the experimental data with those predicted by the models (Figure S4a,c; Table S10). However, none of the independent variables had a statistically significant effect (*p*-value > 0.05) on the DPPH, FRAP, and TPC results (Table 3 and Table S8), so the three models had low significance.

#### 2.2.3. Ultrasonic Bath-Assisted Extraction

**Figure 2:**
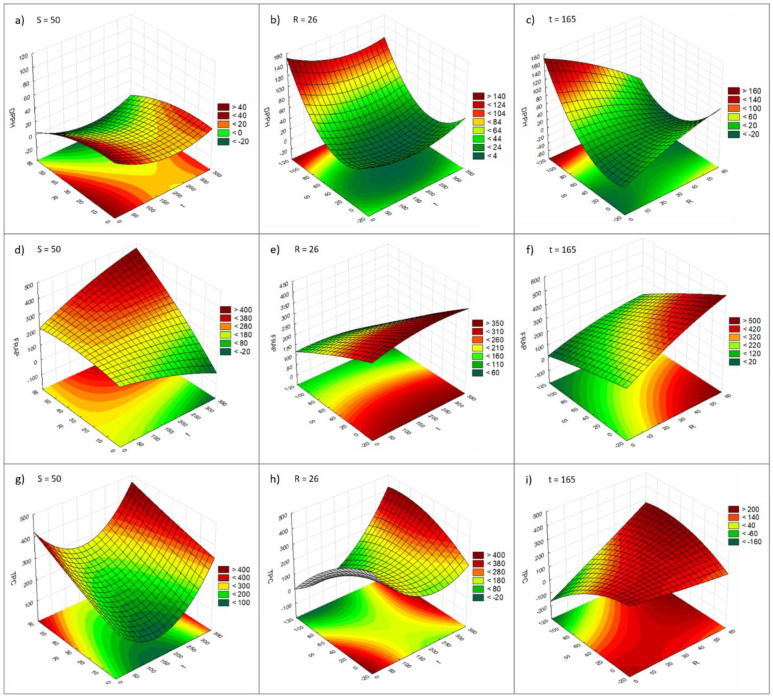
Response surfaces observed for DPPH (**a**–**c**), FRAP (**d**–**f**), and TPC (**g**–**i**) for ultrasonic bath-assisted extraction. DPPH, FRAP, and TPC results are presented as IC_50_ (µg/mL), µmol AAE/g extract, and mg GAE/g extract, respectively. **t** (s); **R** (g biomass/100 mL solvent); **S** (% water in a water/ethanol mixture).

For the ultrasonic bath-assisted extractions, the model relating the combined effects of the independent variables **t**, **R**, and **S** on the DPPH response values showed low significance and a poor fit to the experimental data (*R*^2^ 0.739, *R*^2^ *adj.* 0.348; Table 3 and Table S9). Variable **S** exerted a positive linear effect and a positive quadratic effect on the DPPH results, and no correlation between independent variables was statistically significant (*p*-value > 0.05, Table 3 and Table S10). As for the regression coefficients in the model, none were statistically significant (*p*-value > 0.05), so no equation is described for Y_DPPH_. When comparing the experimental results with those predicted by the model, large residuals were obtained, revealing a poor fit of the model to the experimental data (Figure S6a; Table S14). In general, the IC_50_ value was lower with higher **R** and lower **S,** while variable **t** had no significant effect on the DPPH result (Figure 2a–c).

For the FRAP test, the model (Equation (2), Table 3) showed high significance and a good fit with the experimental data (*R*^2^ 0.942, *R*^2^ *adj.* 0.855; Table 3 and Table S9). As was observed for vortex-assisted extraction, variable **R** had the greatest influence on the results, with a positive linear effect and a positive quadratic effect, while variable **S** had a negative linear effect (*p <* 0.05, Table 3 and Table S10). The correlations between variables **t** and **R**, and between variables **R** and **S** were also statistically significant (*p <* 0.05, Table 3 and Table S10). When comparing the experimental results with those predicted by the model, small residuals were obtained, revealing a good fit of the model to the experimental data (Figure S6b; Table S14). An increase in the % of water in the extraction solvent (**S**) from 0 to 100% led to a 61% decrease in the FRAP result (t = 300 s, R = 50 g/100 mL), while an increase in the biomass/solvent ratio (**R**) from 2 to 50 g/100 mL solvent led to a 79% increase in the FRAP result (t = 300 s, S = 0%). In general, higher **t** and **R** and lower **S** resulted in higher FRAP values (Figure 2d–f), similar to what was observed for the DPPH results.

With regard to the TPC assay, the model describing the combined effects of the independent variables **t**, **R**, and **S** on the TPC response values (Equation (3), Table 3) showed high significance, but a poor fit to the experimental data (*R*^2^ 0.614, *R*^2^ *adj.* 0.034; Table 3 and Table S9). When comparing the experimental results with those predicted by the model, large residuals were obtained, revealing a poor fit of the model to the experimental data (Figure S6c; Table S14). All the independent variables and their combinations had a significant effect on the TPC result (*p <* 0.05, Table 3 and Table S10). Variables **t** and **R** had positive linear and quadratic effects, while variable S had a negative linear and quadratic effect on the TPC assay results (*p <* 0.05, Table 3 and Table S10). Variables **t** and **S** affected the TPC results the most, while the combined effects of the variables had a slight influence. An increase in the % of water in the extraction solvent (**S**) from 0 to 100% led to a 55% increase in the TPC result (t = 300 s, R = 50 g/100 mL), while an increase in the biomass/solvent ratio (**R**) from 2 to 50 g/100 mL of solvent led to a 62% increase in the TPC result. In general, the TPC value increases with higher **R** and **t** and lower **S** (Figure 2g–i). 

#### 2.2.4. Ultrasonic Probe-Assisted Extraction

As for the ultrasonic probe-assisted extractions, the models describing the combined effects of the independent variables **t**, **R**, and **S** on the DPPH and FRAP response values showed a good fit to the experimental data (*R*^2^ 0.831, *R*^2^ *adj.* 0.578 for the DPPH model; *R*^2^ 0.830, *R*^2^ *adj.* 0.575 for the FRAP model; Table 3 and Table S13), while the model for the TPC response showed a poor fit to the experimental data (*R*^2^ 0.602, *R*^2^ *adj.* 0.005; Table 3 and Table S13). When comparing the experimental data with those predicted by the models, an average/good fit was observed for the DPPH and FRAP models (Figure S8a,b; Table S18) and a poor fit was observed for the TPC model (Figure S8c; Table S18). However, as was observed for the extractions with the Ultra-Turrax^®^ homogenizer, none of the independent variables had a statistically significant effect on the DPPH, FRAP, and TPC results (Table 3 and Table S14), so the three models had low significance.

#### 2.2.5. High-Pressure-Assisted Extraction

**Figure 3:**
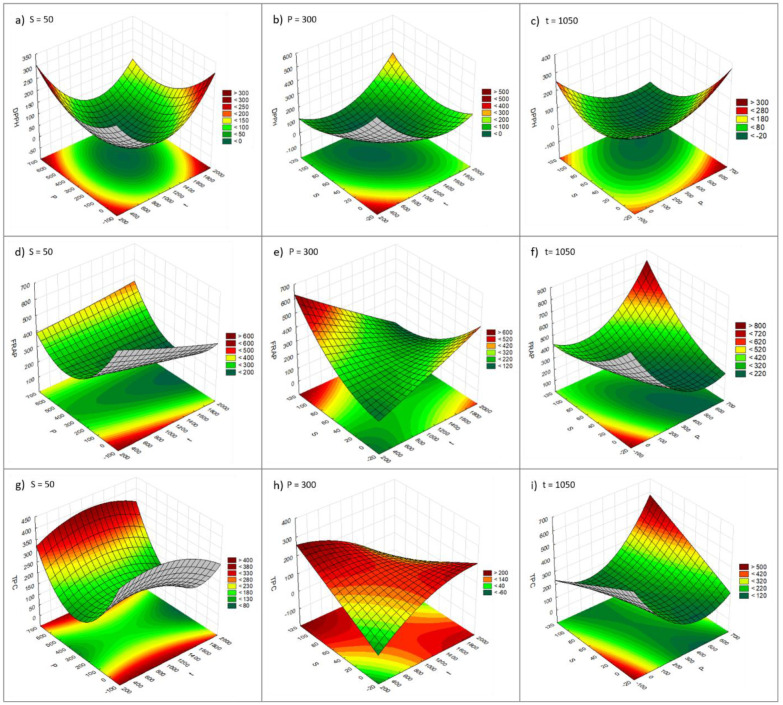
Response surfaces observed for DPPH (**a**–**c**), FRAP (**d**–**f**), and TPC (**g**–**i**) for the high-pressure assisted extraction. DPPH, FRAP, and TPC results are presented as IC_50_ (µg/mL), µmol AAE/g extract, and mg GAE/g extract, respectively. **t** (s); **P** (MPa); **S** (% water in a water/ethanol mixture).

Concerning the high-pressure-assisted extraction, the model describing the combined effects of the independent variables **t**, **P**, and **S** on the DPPH response values (Equation (4), Table 3) showed high significance (*p* < 0.05, Table 3 and Table S16), but a poor fit to the experimental data (*R*^2^ 0.520, *R*^2^ *adj.* 0.087; Table 3 and Table S15). All three variables **t**, **P**, and **S** exerted a negative linear effect and a positive quadratic effect on the DPPH assay results (*p <* 0.05, Table 3 and Table S16). The correlations between the variables **t** and **P**, **t** and **S**, and **P** and **S** were also statistically significant (*p <* 0.05, Table 3 and Table S16). However, when comparing the experimental results with those predicted by the model, large residuals were obtained, revealing a poor fit of the model to the experimental data (Figure S10a; Table S22). In general, lower IC_50_ values were obtained with medium/high extraction times, pressures, and water % studied (Figure 3a–c). 

For the FRAP assay, the model (Equation (5), Table 3) showed low significance, as only variable **P** showed a significant effect, with a positive quadratic effect (*p <* 0.05, Table 3 and Table S16). The model also showed a poor fit with the experimental results (*R*^2^ 0.775, *R*^2^ *adj.* 0.573; Table 3 and Table S15). Comparing the experimental results with those predicted by the model, medium/small residues were obtained, indicating a reasonable fit of the model to the experimental data (Figure S10b; Table S22). In general, higher FRAP values can be obtained with higher **P** and **S** and lower **t** (Figure 3d–f). 

With regard to the TPC assay, the results were very similar to those obtained for the FRAP assay. The model for TPC (Equation (6), Table 3) also showed low significance, with only variable **P** exerting a significant effect (positive quadratic effect) and no correlation between the variables were significant (*p*-value > 0.05, Table 3 and Table S16). The model also showed a poor fit to the experimental results (*R*^2^ 0.705, *R*^2^ *adj.* 0.440; Table 3 and Table S15). When comparing the experimental results with those predicted by the model, large residuals were obtained, which indicate a poor fit of the model to the experimental data (Figure S10c; Table S22). In general, higher TPC values are obtained with high **P** combined with high **S** or low **P** combined with low **S**, while the extraction time does not seem to have a significant effect on the TPC result (Figure 3g–i). 

### 2.3. Effect of **t**, **R**, **S**, and **P** on the Phenolic Content and Antioxidant Capacity of the Extracts 

Analyzing the effect of extraction time (**t**), biomass/solvent ratio (**R**), and water % in water/ethanol mixture (**S**) on TPC and antioxidant capacity (DPPH and FRAP) of the extracts, it can be concluded that, in general, the highest TPC and antioxidant capacity are obtained with higher **R** and **t**, and lower **S** (higher ethanol %) for extractions using a vortex, ultrasonic probe, ultrasonic bath, or Ultra-Turrax^®^ homogenizer. These results are in line with those described in the literature. Hassan et al. [25] also observed that the TPC value increased with extraction time, when analyzing five Vietnamese brown macroalgae using ultrasound-assisted extraction. However, they found that both an increase in the concentration of ethanol in the ethanol/water mixture and an increase in biomass/solvent ratio led to a decrease in TPC, unlike the present study. In a study of brown algae, Fu et al. (2016) [26] observed an increase in the quantity of phenolic compounds and antioxidant capacity with ethanol concentration, up to certain concentrations (up to 50–75%), using conventional extraction. They also observed the same behavior with an increase in the biomass/solvent ratio (up to 10 g/100 mL). In relation to the extraction time, they observed that either the extraction time had no effect on the quantity of phenolic compounds and antioxidant capacity, or they increased up a certain extraction time, depending on the algae. Dang et al. [24] also observed an increase in phenolic content and antioxidant capacity with extraction time in extracts obtained by ultrasound-assisted extraction with 70% ethanol. 

Regarding the effect of pressure (**P**) on the phenolic content and antioxidant capacity, higher TPC and antioxidant capacity (lower IC_50_ in DPPH and higher FRAP) were obtained with higher pressures in the high-pressure-assisted extraction. Heffernan et al. [27] reported that *Fucus serratus* extracts obtained with ethanol/water (80/20) by pressurized-liquid extraction presented lower TPC and lower antioxidant activities than the extracts obtained by solid–liquid extraction, although higher extraction yields were obtained with pressurized extraction. Tierney et al. [23] also found that traditional solid–liquid extraction was more effective for extracting polyphenols from *F. spiralis* with ethanol/water (80/20) than pressurized liquid extraction. Similarly, extracts with higher DPPH and FRAP were obtained for the conventional extraction. These results are contrary to those obtained in the present study. However, unlike high-pressure extraction, pressurized liquid extraction uses high temperatures, which may have caused some degradation of the compounds with antioxidant capacity in the study by Heffernan et al. [27]. 

### 2.4. Selection of the Best Conditions

One of the aims of this study was to optimize the extraction conditions to obtain the maximum content of antioxidant compounds. Knowing the individual and combined effects that each independent variable has on extraction, a model was built to predict the optimum conditions under which the highest TPC content and antioxidant capacity (higher FRAP values and lower DPPH values) are obtained. The optimum conditions were determined by maximizing the desirability of the responses, i.e., the maximum TPC and FRAP and minimum DPPH. For each extraction method, the optimum extraction conditions were obtained for an optimum TPC, FRAP, and DPPH response, individually and together. The results are shown in Table 4. 

Analyzing Table 4, it can be concluded that it was not possible to find an extraction method and a set of optimum experimental conditions at which both higher TPC and antioxidant capacity (higher FRAP values and lower DPPH values) could be obtained. This may due the different specificity of the assays used to quantify phenolic compounds and assess antioxidant capacity. 

For the selection of the optimal conditions, it was considered that the model relating the combined effects of the independent variables **t**, **R**, and **S** on the FRAP response values for vortex-assisted extraction was the one that best fitted the experimental data (Equation (1), R^2^ 0.957 and Equation (2), R^2^ 0.942, respectively; Table 3). The optimum conditions for maximum FRAP in vortex-assisted extraction (t = 300 s, R = 50 g and S = 25%, Table 4) were therefore chosen to validate the model. Table 5 shows the predicted and experimental results for DPPH, FRAP, and TPC analyzed from extracts obtained with the optimum extraction conditions, using vortex-assisted extraction. The DPPH value obtained for the optimum conditions was lower than the value predicted by the model, but within the confidence interval. The experimental TPC value obtained was in agreement with the one predicted by the model and was within the confidence interval. However, the experimental value obtained for the FRAP assay was lower than that predicted by the model and outside the confidence interval, despite the good fit of the model. One possible explanation for this could be the time interval between the FRAP assays for the optimization and for the optimum conditions. Although this time interval was the same for the TPC, FRAP, and DPPH assays, it may be that the compounds responsible for FRAP activity are more susceptible to degradation over time.

## 3. Materials and Methods

### 3.1. Biomass

Brown macroalgae *Fucus spiralis* was collected in the summer of 2018 in the upper intertidal zone of the Marques Neves beach (39°22′13.5″ N 9°23′14.8″ W), Peniche (Portugal) during low tide. The algae were washed with saltwater to remove invertebrates and other organisms, sand, and debris, frozen at −80 °C, and freeze-dried (Scanvac Cool Safe, LaboGene, Lillerod, Denmark). Freeze-dried *F. spiralis* was finely ground using a laboratory blender (Bimby, Vorwerk, Thermomix 31-1, Wuppertal, Germany) and stored in a dry, light-free container until further studies.

### 3.2. Chemicals

HCl 37%, absolute ethanol, methanol, and dimethyl sulfoxide (DMSO) were obtained from commercial suppliers. Folin–Ciocalteu reagent, gallic acid, DPPH (2,2-difenil-1-picrilhidrazil), TPTZ (2,4,6-Tri(2-pyridyl)-s-triazine), FECl_3_, sodium acetate, sodium carbonate, and acetic acid were purchased from Sigma-Aldrich (St. Louis, MO, USA). 

### 3.3. Extraction Procedures

Different extraction methods and conditions were tested. The extraction methods used were ultrasound-assisted extraction (ultrasonic bath and ultrasonic probe), extraction with a vortex, extraction with an Ultra-Turrax^®^ homogenizer, and high-pressure-assisted extraction. The extraction conditions evaluated were extraction time (**t**, 30–300 s), biomass/solvent ratio (**R**, 2–50 g biomass/100 mL solvent), solvent (**S**, water % in water/ethanol mixture, 0–100 % water), and pressure (**P**, 0.1–600 MPa). The experiment design was defined using RSM. 

For all extraction methods, the biomass was suspended in a water/ethanol mixture and the extraction method was applied for a certain period. After extraction, the suspension was filtered and the residue discarded. The solvent was removed under reduced pressure using a rotary evaporator. The dried extracts were dissolved in DMSO at known concentrations and frozen at −20 °C until further analysis. 

#### 3.3.1. Ultrasound-Assisted Extraction

The biomass suspension was placed either in an ultrasonic bath (Ultrasonic Cleaner, VWR USC-TH, Radnor, PA, USA) or a sonicator (S2500 Branson Digital Sonicator, Danbury, CT, USA, 10% amplitude) for different periods. In the latter case, the sample was cooled in an ice bath to avoid overheating and a 30/20 s “on and off” ultrasonic pulse cycle was used.

#### 3.3.2. Vortex Extraction

Samples of the biomass suspension were mixed using a vortex (Vortex Mixer VV3, VWR, Darmstadt, Germany) for different periods.

#### 3.3.3. Extraction with Ultra-Turrax^®^ Homogenizer

Biomass suspension samples were mixed using an Ultra-Turrax^®^ homogenizer (T18 basic, IKA, Staufen, Germany) for different lengths of time.

#### 3.3.4. High-Pressure-Assisted Extraction

Samples of the biomass suspension were placed in vacuum-sealed polyamide/polyethylene (PA/PE) bags and extraction was carried out in industrial-scale high-pressure equipment (Model 55, Hyperbaric, Burgos, Spain) for different time intervals at different pressure levels.

### 3.4. TPC—Total Phenolic Content

The TPC of the *F. spiralis* extracts was determined using the Folin–Ciocalteu method adapted from Singleton and Rossi [28]. A quantity of 2 µL of sample solution in DMSO was added to a mixture of 158 µL of distilled water, 10 µL of Folin–Ciocalteu reagent and 30 µL of a 20% sodium carbonate solution. After 1 h of reaction in the dark, absorbance was measured at 755 nm (Synergy H1 Multi-Mode Microplate Reader, Biotek^®^ Instruments, Winooski, VT, USA). Gallic acid was used as a standard to calculate the phenolic content of the samples and the results are presented as mg of gallic acid equivalents/g of extract (mg GAE/g). 

### 3.5. DPPH Radical Scavenging Activity

The DPPH free radical scavenging method was adapted from Brand-Williams, Cuvelier, and Berset [29]. A solution of 0.1 mM DPPH radicals in methanol was freshly prepared. Various concentrations of 2 mL of sample solution (1, 3, 5, 10, 30, and 50 mg/mL in DMSO) were added to 198 mL of the DPPH radical solution in 96-well microplates. The mixture was left to stand at room temperature in the dark for 30 min, at which point the decrease in absorbance at 517 nm was measured (Synergy H1 Multi-Mode Microplate Reader, BioTek^®^ Instruments, Winooski, VT, USA). The capacity to scavenge the DPPH radical was calculated using Equation (7):

Scavenging effect (%) = [1 − (A_sample_ − A_sample_blank_)/A_control_)] × 100 (7)

where A_control_ is the absorbance of the control (DPPH solution with DMSO), A_sample_ is the absorbance of the test sample (DPPH solution plus test sample), and A_sample_blank_ is the absorbance of the sample in methanol (sample without DPPH solution). The results are presented as IC_50_ values (mg/mL), calculated using GraphPad Prism 5.01 software (GraphPad Software Inc., San Diego, CA, USA).

### 3.6. FRAP—Ferric Reducing Antioxidant Power

The FRAP assay was adapted from Benzie and Strain [30]. The FRAP reagent was prepared by mixing 0.3 M acetate buffer (pH = 3.6), 10 mM TPTZ in 40 mM HCl, and 20 mM aqueous solution FeCl_3_ in a 10:1:1 ratio. A quantity of 5 µL of sample solution was added to 195 µL of FRAP reagent (distilled water for the blank assays) in 96-well microplates. The mixture was left to stand in the dark for 4 h, at which point the increase in absorbance at 593 nm was measured (Synergy H1 Multi-Mode Microplate Reader, BioTek^®^ Instruments, Winooski, VT, USA). Ascorbic acid was used as a standard and the results are presented as µmol ascorbic acid equivalents/g of extract (µmol AAE/g).

### 3.7. Variable Selection and Design of Experiment

RSM was used to optimize the extraction conditions. The independent variables tested were extraction time (**t**, 30–300 s), biomass/solvent ratio (**R**, 2–50 g biomass/100 mL solvent), and solvent (**S**, water % in water/ethanol mixture, 0–100% water), for extractions using a vortex, homogenizer, ultrasonic bath, and ultrasonic probe. A central composite rotatable design (CCRD) was applied, and 16 experimental runs were carried out with different combinations of the three factors (t, R, and S), including two replicated values at the central point. In the high-pressure-assisted extractions, the independent variables studied were extraction time (**t**, 300–1800 s), solvent (**S**, water % in water/ethanol mixture, 0–100% water), and pressure (**P**, 0.1–600 MPa). A circumscribed central composite design (CCCD) was applied and 20 experimental runs were carried out with different combinations of three factors (t, P, and S), including 6 replicates at the central point. DPPH, FRAP, and TPC results (IC_50_, µmol AAE/g and mg GAE/g, respectively) were used as response variables for all extraction methods. The coded and decoded levels of the independent variables can be found in Tables S1 and S2 (Supplementary Material). 

### 3.8. Mathematical Modeling and Statistical Analysis

The experimental design and statistical analysis were carried out using Statistica 10 software (StatSoft, Inc., Minneapolis, MN, USA). 

The experimental data were fitted to a second-order polynomial model in order to obtain regression coefficients (Equation (8)) to describe the effect of the chosen parameters and their interactions on the responses.

(8) $$Y=b0+\sum_{i=1}^{k}biXi+\sum_{i=1}^{k}biiX^{2}+\sum_{\begin{array}{c} i=1\\ j>1 \end{array}}^{k-1}\sum_{j=2}^{k}bijXiXj+C$$

where *b*0, *bi*, *bii*, and *bij* are the constant coefficients of the intercept, linear, quadratic, and interaction terms, respectively; *Xi* and *Xj* represent the independent variables (X_t_, X_R_, X_S_, X_P_); *Y* is the predicted response (Y_TCP_, Y_FRAP_, Y_DPPH_); *C* is the residual associated with the experiments; *k* is the number of variables; and *C* is the residual term.

The significance of the model equations, the individual parameters, and factor interaction was evaluated by the analysis of variance (ANOVA) at a confidence interval (CI) of 95% (*p*-value ≤ 0.05). Two-dimensional (2D) contour plots and three-dimensional (3D) surface responses were obtained after applying the quadratic models developed.

The adequacy of the models was determined using the lack-of-fit test and *R*^2^ (coefficient of determination) analysis. Where applicable, results are presented as mean ± standard deviation (SD).

The optimal extraction conditions were determined by maximizing the desirability of the responses (maximum TPC and FRAP and minimum DPPH). Optimization was carried out for each response individually (Y_TCP_, Y_FRAP_, Y_DPPH_) and for the three response parameters (Y_TCP_ + Y_FRAP_ + Y_DPPH_). To validate the model, extractions were carried out using the optimum conditions and the experimental values were compared with those predicted by the model.

## 4. Conclusions

This study shows that the use of different extraction methods and conditions affects the TPC and the antioxidant activity of the resulting extracts. The extracts with the highest TPC values were obtained with ultrasonic probe-assisted extraction, ranging from 94.78 to 474.16 mg GAE/g of extract. Regarding the antioxidant activity of the extracts, the best DPPH results were obtained with vortex-assisted extraction, with IC_50_ values ranging from 1.89 to 16 µg/mL, while the best FRAP results were obtained using Ultra-Turrax^®^ homogenizer extraction, with values ranging from 502.16 to 1188.81 μmol AAE/g of extract.

RSM was used to analyze the combined effects of extraction variables on the total phenolic content and on the antioxidant capacity of *F. spiralis* extracts. In general, higher TPC and antioxidant capacity were obtained with higher biomass/solvent ratios, higher extraction times, higher extraction pressures, and higher % ethanol. Only the models relating the combined effects of the independent variables **t**, **R**, and **S** on the FRAP response values for vortex- and ultrasonic bath-assisted extraction showed statistical significance and a good fit to the experimental data. The model with the best fit, the FRAP response for vortex-assisted extraction, was used to predict the optimum extraction conditions (t = 300 s, R = 50 g, and S = 25%), which were tested experimentally to confirm the predictability of the model. The TPC and DPPH values obtained for the optimum conditions were within the confidence interval, while the FRAP value was outside the confidence interval, despite the model’s good fit.

In this study, it can be concluded that it was not possible to optimize the extraction of antioxidants from *F. spiralis*, i.e., it was not possible to find an extraction method and a set of optimum experimental conditions that would allow a higher TPC and greater antioxidant capacity (higher FRAP values and lower DPPH values) to be obtained. However, a good model was obtained for the combined effects of the independent variables **t**, **R**, and **S** on the FRAP response values for vortex-assisted extraction (R^2^ 0.957), and it was possible to determine optimal extraction conditions for this extraction method. 

In future studies, the TPC and antioxidant capacity of the antioxidant-enriched *Fucus spiralis* extracts obtained should be compared with commercial antioxidant food additives in order to assess their potential as natural antioxidant additives for functional foods.

## Figures and Tables

**Table 1:** Results obtained for the different response variables (DPPH, FRAP, and TPC), with the different extraction methods (vortex, Ultra-Turrax^®^ homogenizer, ultrasonic bath, and ultrasonic probe), and under different conditions (runs 1 to 16). The DPPH, FRAP, and TPC results are presented as IC_50_ (µg/mL), µmol AAE/g extract, and mg GAE/g extract, respectively. Each value is presented as mean value (*n* = 3) ± standard deviation.

	Independent Variables			Vortex			Ultra-Turrax^®^ Homogenizer			Ultrasonic Bath			Ultrasonic Probe		
Run	t (s)	R (g)	S (%)	DPPH	FRAP	TPC	DPPH	FRAP	TPC	DPPH	FRAP	TPC	DPPH	FRAP	TPC
1	85	12	20	2.19 ± 0.19	265.06 ± 3.08	100.46 ± 2.86	13.25 ± 0.06	620.74 ± 52.80	189.90 ± 16.99	2.25 ± 0.01	265.10 ± 37.67	290.25 ± 11.27	3.73 ± 0.31	321.27 ± 24.83	291.17 ± 15.26
2	85	12	80	7.51 ± 0.14	157.30 ± 27.06	61.16 ± 4.39	20.24 ± 2.82	858.11 ± 119.49	110.05 ± 20.63	49.25 ± 8.65	157.15 ± 6.44	66.72 ± 1.45	33.18 ± 5.40	215.36 ± 10.27	173.29 ± 24.34
3	85	40	20	2.33 ± 0.16	331.37 ± 19.11	155.63 ± 9.13	11.27 ± 0.73	835.03 ± 44.70	154.89 ± 19.32	11.81 ± 1.27	331.53 ± 15.04	259.84 ± 19.60	6.07 ± 0.87	412.24 ± 60.41	474.16 ± 33.46
4	85	40	80	5.47 ± 1.07	227.70 ± 21.80	65.86 ± 1.46	8.64 ± 1.46	945.63 ± 134.87	157.02 ± 4.18	5.59 ± 0.43	227.67 ± 26.39	170.66 ± 20.99	8.21 ± 0.22	233.05 ± 29.32	213.30 ± 23.12
5	245	12	20	4.13 ± 0.00	208.01 ± 5.30	71.70 ± 11.45	9.77 ± 1.87	971.09 ± 138.90	185.60 ± 3.61	6.47 ± 1.13	207.95 ± 9.92	177.32 ± 9.56	6.43 ± 0.69	399.73 ± 64.34	179.60 ± 17.44
6	245	12	80	6.43 ± 0.39	119.42 ± 8.93	44.07 ± 7.22	28.34 ± 4.74	502.16 ± 80.13	120.16 ± 19.94	39.19 ± 2.14	119.20 ± 8.69	106.57 ± 17.86	20.06 ± 3.85	302.36 ± 59.94	269.45 ± 46.08
7	245	40	20	1.89 ± 0.25	419.45 ± 19.25	176.26 ± 6.87	7.14 ± 0.92	850.46 ± 24.06	185.11 ± 12.43	12.06 ± 0.00	419.76 ± 29.59	154.22 ± 27.72	5.57 ± 1.07	442.01 ± 48.25	408.05 ± 20.69
8	245	40	80	4.13 ± 0.48	253.09 ± 8.01	63.43 ± 9.06	9.10 ± 0.45	1143.08 ± 101.74	157.23 ± 4.28	4.91 ± 0.87	253.10 ± 0.52	170.47 ± 19.73	13.86 ± 1.90	181.79 ± 20.03	94.78 ± 16.12
9	30	26	50	3.78 ± 0.06	218.84 ± 1.35	108.49 ± 8.79	23.67 ± 2.51	1188.81 ± 185.00	121.22 ± 9.63	46.60 ± 0.42	218.79 ± 28.17	197.55 ± 13.50	6.65 ± 0.46	263.77 ± 38.71	211.72 ± 27.07
10	300	26	50	16.00 ± 0.28	200.15 ± 9.36	25.04 ± 4.46	12.70 ± 1.63	820.07 ± 33.70	116.01 ± 17.99	15.90 ± 2.90	200.08 ± 1.08	400.12 ± 32.14	7.14 ± 0.81	352.05 ± 59.94	220.83 ± 36.78
11	165	2	50	11.90 ± 0.71	96.51 ± 5.96	36.48 ± 6.04	20.96 ± 3.77	791.74 ± 51.39	135.98 ± 12.87	20.15 ± 1.87	96.25 ± 6.58	71.03 ± 11.33	18.46 ± 3.44	154.91 ± 29.34	181.25 ± 29.90
12	165	50	50	2.68 ± 0.30	316.70 ± 20.01	86.01 ± 11.85	10.55 ± 1.95	620.53 ± 86.74	123.95 ± 16.66	4.83 ± 0.00	316.83 ± 44.00	321.31 ± 19.63	12.99 ± 2.18	371.83 ± 65.08	435.08 ± 62.97
13	165	26	0	3.78 ± 0.06	325.37 ± 47.01	95.77 ± 7.41	3.67 ± 0.30	1011.33 ± 142.54	249.93 ± 38.65	2.65 ± 0.13	299.70 ± 5.22	171.79 ± 7.93	2.79 ± 0.43	517.85 ± 4.53	262.35 ± 1.22
14	165	26	100	9.97 ± 0.47	139.97 ± 24.35	53.91 ± 6.79	16.89 ± 1.80	718.92 ± 97.12	119.92 ± 10.17	103.55 ± 14.95	139.79 ± 7.64	140.76 ± 13.92	11.14 ± 0.28	215.79 ± 39.54	269.59 ± 12.20
15 (C)	165	26	50	4.18 ± 0.68	248.31 ± 0.09	80.83 ± 9.05	15.88 ± 2.01	798.40 ± 69.88	149.39 ± 21.45	6.15 ± 1.11	248.31 ± 36.77	162.89 ± 20.28	5.14 ± 0.75	463.98 ± 76.80	165.33 ± 17.81
16 (C)	165	26	50	3.75 ± 0.30	250.27 ± 31.50	106.26 ± 20.60	21.28 ± 2.82	568.89 ± 82.02	101.53 ± 3.89	10.09 ± 1.59	250.28 ± 10.94	163.02 ± 16.34	8.41 ± 0.06	286.66 ± 48.55	460.03 ± 62.87

C—Central point.

**Table 2:** Results obtained for the different response variables (DPPH, FRAP, and TPC), using high-pressure assisted extraction under different conditions (runs 1 to 16). DPPH, FRAP, and TPC results are presented as IC_50_ (µg/mL), µmol AAE/g extract, and mg GAE/g extract, respectively. Each value is presented as mean value (*n* = 3) ± standard deviation.

	Independent Variables			High Pressure		
Run	t (s)	P (MPa)	S (%)	DPPH	FRAP	TPC
1	604	122	20	212.83 ± 82.17	324.92 ± 17.87	190.66 ± 0.77
2	1496	122	20	163.45 ± 0.35	329.35 ± 26.56	190.62 ± 2.02
3	604	479	20	300.00 ± 0.00	178.07 ± 20.43	127.89 ± 0.83
4	1496	479	20	64.31 ± 10.80	229.72 ± 43.05	190.42 ± 1.38
5	604	122	80	143.97 ± 41.10	399.01 ± 37.12	193.86 ± 1.22
6	1496	122	80	54.77 ± 6.34	202.42 ± 16.09	111.05 ± 4.47
7	604	479	80	14.24 ± 1.09	409.50 ± 7.42	300.04 ± 5.14
8	1496	479	80	34.50 ± 2.18	305.96 ± 17.05	205.55 ± 1.21
9	300	300	50	53.36 ± 13.71	243.42 ± 0.88	103.45 ± 2.80
10	1800	300	50	78.01 ± 30.04	206.25 ± 18.56	118.88 ± 1.63
11	1050	0	50	4.39 ± 0.43	391.56 ± 32.04	331.42 ± 1.49
12	1050	600	50	4.06 ± 1.68	312.01 ± 46.85	246.45 ± 1.66
13	1050	300	0	12.28 4.06	272.19 ± 27.18	162.99 ± 1.20
14	1050	300	100	10.83 ± 2.31	280.76 ± 14.22	144.92 ± 0.99
15 (C)	1050	300	50	11.11 ± 5.04	186.15 ± 17.83	105.59 ± 1.09
16 (C)	1050	300	50	11.09 ± 0.81	300.31 ± 19.27	190.37 ± 0.86
17 (C)	1050	300	50	6.46 ± 3.75	141.46 ± 10.30	82.16 ± 1.04
18 (C)	1050	300	50	4.06 ± 0.57	281.04 ± 8.25	170.54 ± 1.32
19 (C)	1050	300	50	4.80 ± 0.50	262.40 ± 4.64	223.85 ± 2.07
20 (C)	1050	300	50	3.57 ± 0.58	184.79 ± 19.45	168.55 ± 1.73

C—Central point.

**Table 3:** Effects (*p*-value ≤ 0.05) of the independent variables t, R, P, and S on the DPPH, FRAP, and TPC results, for the different extractions methods and mathematical equations. L, linear effect; Q, quadratic effect; +, positive effect; −, negative effect. *R*^2^, regression coefficient; *R*^2^ *adj.*, adjusted regression coefficient; *ns*, non-significant parameters (*p*-value > 0.05).

Extraction Method	Response Variable	Independent Variables with Significant Effects	Equation	*R* ^2^	*R*^2^ *adj.*
Vortex	DPPH	t(L,+); R(L,−); S(L,+)	*ns*	0.523	0.000
	FRAP	t(Q,−); R(L,+;Q,−); S(L,−) t(L) by R(L) (+); R(L) by S(L) (−)	Y_FRAP_ = 235.285 + 3.571X_R_ − 1.192X_S_ − 0.001X_t_^2^ − 0.036X_R_^2^ + 0.023X_t_X_R_ − 0.022X_R_X_S_ (Equation (1))	0.957	0.892
	TPC	*ns*	*ns*	0.762	0.405
Ultra-Turrax^®^ homogenizer	DPPH	*ns*	*ns*	0.899	0.747
	FRAP	*ns*	*ns*	0.466	0.000
	TPC	*ns*	*ns*	0.857	0.642
Ultrasonic bath	DPPH	S(L,+;Q,+)	*ns*	0.739	0.348
	FRAP	R(L,+;Q,+); S(L,−) t(L) by R(L) (+); R(L) by S(L) (−)	Y_FRAP_ = 227.603 + 3.306X_R_ − 0.031X_R_^2^ + 0.023X_t_X_R_ − 0.022X_R_X_S_ (Equation (2))	0.942	0.855
	TPC	t(L,+;Q,+); R(L,+;Q,+); S(L,−;Q,−) t(L) by R(L) (−); t(L) by S(L) (+); R(L) by S(L) (+)	Y_TPC_ = 428.734 − 2.349X_t_ − 0.195X_R_ − 3.484X_S_ + 0.006X_t_^2^ + 0.005X_R_^2^ − 0.015X_S_^2^ − 0.004X_t_X_R_ + 0.014X_t_X_S_ + 0.065X_R_X_S_ (Equation (3))	0.614	0.034
Ultrasonic probe	DPPH	*ns*	*ns*	0.831	0.578
	FRAP	*ns*	*ns*	0.830	0.575
	TPC	*ns*	*ns*	0.602	0.005
High pressure	DPPH	t(L,−;Q,+); P(L,−;Q,+); S(L,−;Q,+) t(L) by P(L) (−); t(L) by S(L) (+); P(L) by S(L) (−)	Y_DPPH_ = 501.12 − 0.55X_t_ − 0.14X_P_ − 4.84X_S_ + 0.001X_P_^2^ + 0.025X_S_^2^ + 0.002X_t_X_S_ − 0.003X_P_X_S_ (Equation (4))	0.520	0.087
	FRAP	P(Q,+)	Y_FRAP_ = 494.823 − 1.640X_P_ + 0.002X_P_^2^ (Equation (5))	0.775	0.573
	TPC	P(Q,+)	Y_TPC_ = 207.798 − 1.286X_P_ + 0.001X_P_^2^ (Equation (6))	0.705	0.440

Note: Only statistically significant regression coefficients were included in the model equations.

**Table 4:** Optimum extraction conditions and predicted TPC, FRAP, and DPPH response values.

Extraction Method	Optimal Conditions			Predicted		
	t (s)	R (g)	S (%)	DPPH	FRAP	TPC
Vortex	165	50	25	1.36		
	300	50	25		454.64	
	300	50	0			193.86
	165	50	0	0.51	457.62	197.35
Ultra-Turrax^®^ homogenizer	300	2	100	33.20		
	300	50	100		1246.87	
	300	14	0			267.53
	165	26	50	18.68	686.38	124.32
Ultrasonic bath	300	50	75	2.14		
	300	50	25		455.44	
	300	50	100			431.29
	165	26	50	9.79	245.63	167.38
Ultrasonic probe	30	14	100	35.42		
	165	50	0		534.31	
	165	50	25			517.81
	165	26	50	6.57	374.88	312.44
	t (s)	P (MPa)	S (%)	DPPH	FRAP	TPC
High pressure	1050	600	100	0		
	1800	600	100		431.12	
	1425	600	100			341.19
	1050	600	100	0	540.66	391.06

**Table 5:** Predicted and experimental response values obtained for vortex-assisted extraction for the optimum extraction conditions. DPPH, FRAP, and TPC results are presented as IC_50_ (µg/mL), µmol AAE/g extract, and mg GAE/g extract, respectively. Each value is presented as the mean value (*n* = 3) ± standard deviation.

Optimal Conditions			Predicted			Experimental		
t (s)	R (g)	S (%)	DPPH	FRAP	TPC	DPPH	FRAP	TPC
300	50	25	6.82	454.64	147.98	2.42 ± 0.35	324.54 ± 15.17	141.92 ± 13.66

## Data Availability

Data are contained within the article and Supplementary Materials.

## Supplementary Materials

The following supporting information can be downloaded at: https://www.mdpi.com/article/10.3390/molecules29102271/s1.
