# Supplementary material for: Extraction of Antioxidants from Brown Macroalgae Fucus spiralis
Source: Molecules. 2024 May 11;29(10):2271. doi: 10.3390/molecules29102271 (PMC11124032; doi:10.3390/molecules29102271)
Supplement: Supplementary file 1 [file molecules-29-02271-s001.zip › molecules-2961320-supplementary.pdf]

Extraction of Antioxidants from Brown Macroalgae *Fucus spiralis*

André Horta [1,2,3,†], Ana M. Duarte [1,†], Sónia Barroso [1,*], Filipa R. Pinto [1], Susana Mendes [1], Vasco Lima [4], Jorge A. Saraiva [4] and Maria M. Gil [1,*]

[1] MARE—Marine and Environmental Sciences Centre/ARNET—Aquatic Research Network, School of Tourism and Maritime Technology, Polytechnic of Leiria, Cetemares, 2520-620 Peniche, Portugal; filipa.pinto@ipleiria.pt (F.R.P.)
[2] Division of Aquaculture, Upgrading and Bioprospection, Portuguese Institute for the Sea and Atmosphere (IPMA), Avenida Magalhães Ramalho, 6, 1495-165 Lisboa, Portugal
[3] Research Institute for Medicines, Faculty of Pharmacy, University of Lisbon, Avenida Professor Gama Pinto, 1649-003 Lisbon, Portugal
[4] LAQV-REQUIMTE, Department of Chemistry, University of Aveiro, 3810-193 Aveiro, Portugal
* Correspondence: sonia.barroso@ipleiria.pt (S.B.); maria.m.gil@ipleiria.pt (M.M.G.)
† These authors contributed equally to this work.

Table of Contents

1. Experimental design and Response Surface Methodology (RSM)

2. Statistical analysis

2.1. Vortex

2.2. Ultra-Turrax® homogenizer

2.3. Ultrasonic bath

2.4. Ultrasonic probe

2.5. High pressure

1. **Experimental design and Response Surface Methodology (RSM)**

Table S1. Coded and decoded levels of the independent variables used for the extraction of antioxidants from *Fucus spiralis*, with the different extraction methods (vortex, Ultra-Turrax® homogenizer, ultrasonic bath and ultrasonic probe).

	**Coded levels**			**Decoded Conditions**		
**Run**	**A**	**B**	**C**	**t (s)**	**R (g)**	**S (%)**
**1**	-1.00000	-1.00000	-1.00000	84,7	11,7	20,3
**2**	-1.00000	-1.00000	1.00000	84,7	11,7	79,7
**3**	-1.00000	1.00000	-1.00000	84,7	40,3	20,3
**4**	-1.00000	1.00000	1.00000	84,7	40,3	79,7
**5**	1.00000	-1.00000	-1.00000	245,3	11,7	20,3
**6**	1.00000	-1.00000	1.00000	245,3	11,7	79,7
**7**	1.00000	1.00000	-1.00000	245,3	40,3	20,3
**8**	1.00000	1.00000	1.00000	245,3	40,3	79,7
**9**	-1.68179	0.00000	0.00000	30,0	26,0	50,0
**10**	1.68179	0.00000	0.00000	300,0	26,0	50,0
**11**	0.00000	-1.68179	0.00000	165,0	2,0	50,0
**12**	0.00000	1.68179	0.00000	165,0	50,0	50,0
**13**	0.00000	0.00000	-1.68179	165,0	26,0	0,0
**14**	0.00000	0.00000	1.68179	165,0	26,0	100,0
**15 (C)**	0.00000	0.00000	0.00000	165,0	26,0	50,0
**16 (C)**	0.00000	0.00000	0.00000	165,0	26,0	50,0

C – Central point

Table S2. Coded and decoded levels of the independent variables used for the extraction of antioxidants from *Fucus spiralis*, using high pressure.

	Coded levels			Decoded Conditions		
**Run**	**A**	**B**	**C**	**t (s)**	**P (MPa)**	**S (%)**
**1**	-1,00	-1,00	-1,00	603,6	121,5	20,2
**2**	1,00	-1,00	-1,00	1496,4	121,5	20,2
**3**	-1,00	1,00	-1,00	603,6	478,6	20,2
**4**	1,00	1,00	-1,00	1496,4	478,6	20,2
**5**	-1,00	-1,00	1,00	603,6	121,5	79,8
**6**	1,00	-1,00	1,00	1496,4	121,5	79,8
**7**	-1,00	1,00	1,00	603,6	478,6	79,8
**8**	1,00	1,00	1,00	1496,4	478,6	79,8
**9**	-1,68	0,00	0,00	300,0	300,1	50,0
**10**	1,68	0,00	0,00	1800,0	300,1	50,0
**11**	0,00	-1,68	0,00	1050,0	0,1	50,0
**12**	0,00	1,68	0,00	1050,0	600,0	50,0
**13**	0,00	0,00	-1,68	1050,0	300,1	0,0
**14**	0,00	0,00	1,68	1050,0	300,1	100,0
**15 (C)**	0,00	0,00	0,00	1050,0	300,1	50,0
**16 (C)**	0,00	0,00	0,00	1050,0	300,1	50,0
**17 (C)**	0,00	0,00	0,00	1050,0	300,1	50,0
**18 (C)**	0,00	0,00	0,00	1050,0	300,1	50,0
**19 (C)**	0,00	0,00	0,00	1050,0	300,1	50,0
**20 (C)**	0,00	0,00	0,00	1050,0	300,1	50,0

C – Central point

2. Statistical analysis

2.1. Vortex

Table S3. Analysis of variance (ANOVA) of the second-order polynomial model for optimization of the extraction of antioxidants from *F. spiralis*, using vortex-assisted extraction. t: extraction time (s); R: biomass:solvent ratio (g biomass/100 mL solvent); S: solvent (% of water in water:ethanol mixture); SS: Sum of squares; df: Degree of freedom; MS: Mean square; F: F value; p: probability; L: Linear effect; Q: Quadratic effect; 1Lby2L: interaction between the linear effect of t and the linear effect of R; 1Lby3L: interaction between the linear effect of t and the linear effect of S; 2Lby3L: interaction between the linear effect of R and the linear effect of S. Significant parameters are presented in red.

	DPPH					*FRAP*					*TPC*				
	SS	df	MS	F	p	SS	df	MS	F	p	SS	df	MS	F	p
(1)t (L)	28,23	1	28,23	305,35	0,036	12,2	1	12,17	6,31	0,241	2066,60	1	2066,60	6,39	0,240
t (Q)	12,49	1	12,49	135,15	0,055	365,7	1	365,71	189,50	0,046	103,79	1	103,79	0,32	0,672
(2)R (L)	35,25	1	35,25	381,29	0,033	53168,1	1	53168,14	27550,51	0,004	5224,04	1	5224,04	16,16	0,155
R (Q)	0,55	1	0,55	5,92	0,248	494,4	1	494,40	256,18	0,040	260,09	1	260,09	0,80	0,535
(3)S (L)	40,15	1	40,15	434,25	0,031	44342,4	1	44342,44	22977,24	0,004	8461,26	1	8461,26	26,18	0,123
S (Q)	0,08	1	0,08	0,89	0,518	33,8	1	33,82	17,52	0,149	2,24	1	2,24	0,01	0,947
1L by 2L	0,87	1	0,87	9,46	0,200	5428,4	1	5428,37	2812,86	0,012	512,81	1	512,81	1,59	0,427
1L by 3L	1,93	1	1,93	20,83	0,137	236,8	1	236,78	122,70	0,057	16,21	1	16,21	0,05	0,860
2L by 3L	0,62	1	0,62	6,75	0,234	678,7	1	678,70	351,69	0,034	2300,23	1	2300,23	7,12	0,228
Lack of Fit	110,74	5	22,15	239,57	0,049	4720,8	5	944,17	489,25	0,034	5580,00	5	1116,00	3,45	0,386
Pure Error	0,09	1	0,09			1,9	1	1,93			323,20	1	323,20		
Total SS	232,34	15				109634,6	15				24811,34	15			
R^2			0,523					0.957					0.762		
R^2 *adj.*			0.000					0.892					0.405		

Table S4. Effect estimates of variables in vortex-assisted extraction. t: extraction time (s); R: biomass:solvent ratio (g biomass/100 mL solvent); S: solvent (% of water in water:ethanol mixture); L: Linear effect; Q: Quadratic effect; 1Lby2L: interaction between the linear effect of t and the linear effect of R; 1Lby3L: interaction between the linear effect of t and the linear effect of S; 2Lby3L: interaction between the linear effect of R and the linear effect of S. Significant parameters are presented in red.

	Parameter	Effect	Std.Err. - Pure Err	t(1)	p	-95,% - Cnf.Limt	+95,% - Cnf.Limt	Coeff.	Std.Err. - Coeff.	-95,% - Cnf.Limt	+95,% - Cnf.Limt
DPPH	Mean/Interc.	4,35	0,21	20,30	0,031	1,63	7,08	4,35	0,21	1,63	7,08
	(1)t (L)	2,88	0,16	17,47	0,036	0,78	4,97	1,44	0,08	0,39	2,48
	t (Q)	2,32	0,20	11,63	0,055	-0,22	4,86	1,16	0,10	-0,11	2,43
	(2)R (L)	-3,21	0,16	-19,53	0,033	-5,30	-1,12	-1,61	0,08	-2,65	-0,56
	R (Q)	0,49	0,20	2,43	0,248	-2,05	3,02	0,24	0,10	-1,03	1,51
	(3)S (L)	3,43	0,16	20,84	0,031	1,34	5,52	1,71	0,08	0,67	2,76
	S (Q)	0,19	0,20	0,95	0,518	-2,35	2,73	0,09	0,10	-1,17	1,36
	1L by 2L	-0,66	0,22	-3,08	0,200	-3,39	2,07	-0,33	0,11	-1,70	1,04
	1L by 3L	-0,98	0,22	-4,56	0,137	-3,71	1,75	-0,49	0,11	-1,86	0,88
	2L by 3L	-0,56	0,22	-2,60	0,234	-3,29	2,17	-0,28	0,11	-1,65	1,09
FRAP	Mean/Interc.	246,06	0,98	251,23	0,003	233,62	258,51	246,06	0,98	233,62	258,51
	(1)t (L)	-1,89	0,75	-2,51	0,241	-11,44	7,67	-0,94	0,38	-5,72	3,83
	t (Q)	-12,57	0,91	-13,77	0,046	-24,17	-0,97	-6,28	0,46	-12,08	-0,48
	(2)R (L)	124,79	0,75	165,98	0,004	115,24	134,34	62,40	0,38	57,62	67,17
	R (Q)	-14,61	0,91	-16,01	0,040	-26,21	-3,01	-7,31	0,46	-13,10	-1,51
	(3)S (L)	-113,96	0,75	-151,58	0,004	-123,52	-104,41	-56,98	0,38	-61,76	-52,21
	S (Q)	3,82	0,91	4,19	0,149	-7,78	15,42	1,91	0,46	-3,89	7,71
	1L by 2L	52,10	0,98	53,04	0,012	39,62	64,58	26,05	0,49	19,81	32,29
	1L by 3L	-10,88	0,98	-11,08	0,057	-23,36	1,60	-5,44	0,49	-11,68	0,80
	2L by 3L	-18,42	0,98	-18,75	0,034	-30,90	-5,94	-9,21	0,49	-15,45	-2,97
TPC	*Mean/Interc.*	91,01	12,67	7,18	0,088	-70,04	252,06	91,01	12,67	-70,04	252,06
	(1)t (L)	-24,60	9,73	-2,53	0,240	-148,23	99,02	-12,30	4,86	-74,11	49,51
	t (Q)	-6,69	11,81	-0,57	0,672	-156,79	143,41	-3,35	5,91	-78,40	71,70
	(2)R (L)	39,12	9,73	4,02	0,155	-84,51	162,74	19,56	4,86	-42,25	81,37
	R (Q)	-10,60	11,81	-0,90	0,535	-160,70	139,50	-5,30	5,91	-80,35	69,75
	(3)S (L)	-49,78	9,73	-5,12	0,123	-173,41	73,84	-24,89	4,86	-86,70	36,92
	S (Q)	-0,98	11,81	-0,08	0,947	-151,08	149,12	-0,49	5,91	-75,54	74,56
	1L by 2L	16,01	12,71	1,26	0,427	-145,51	177,54	8,01	6,36	-72,76	88,77
	1L by 3L	-2,85	12,71	-0,22	0,860	-164,37	158,68	-1,42	6,36	-82,19	79,34
	2L by 3L	-33,91	12,71	-2,67	0,228	-195,44	127,61	-16,96	6,36	-97,72	63,81

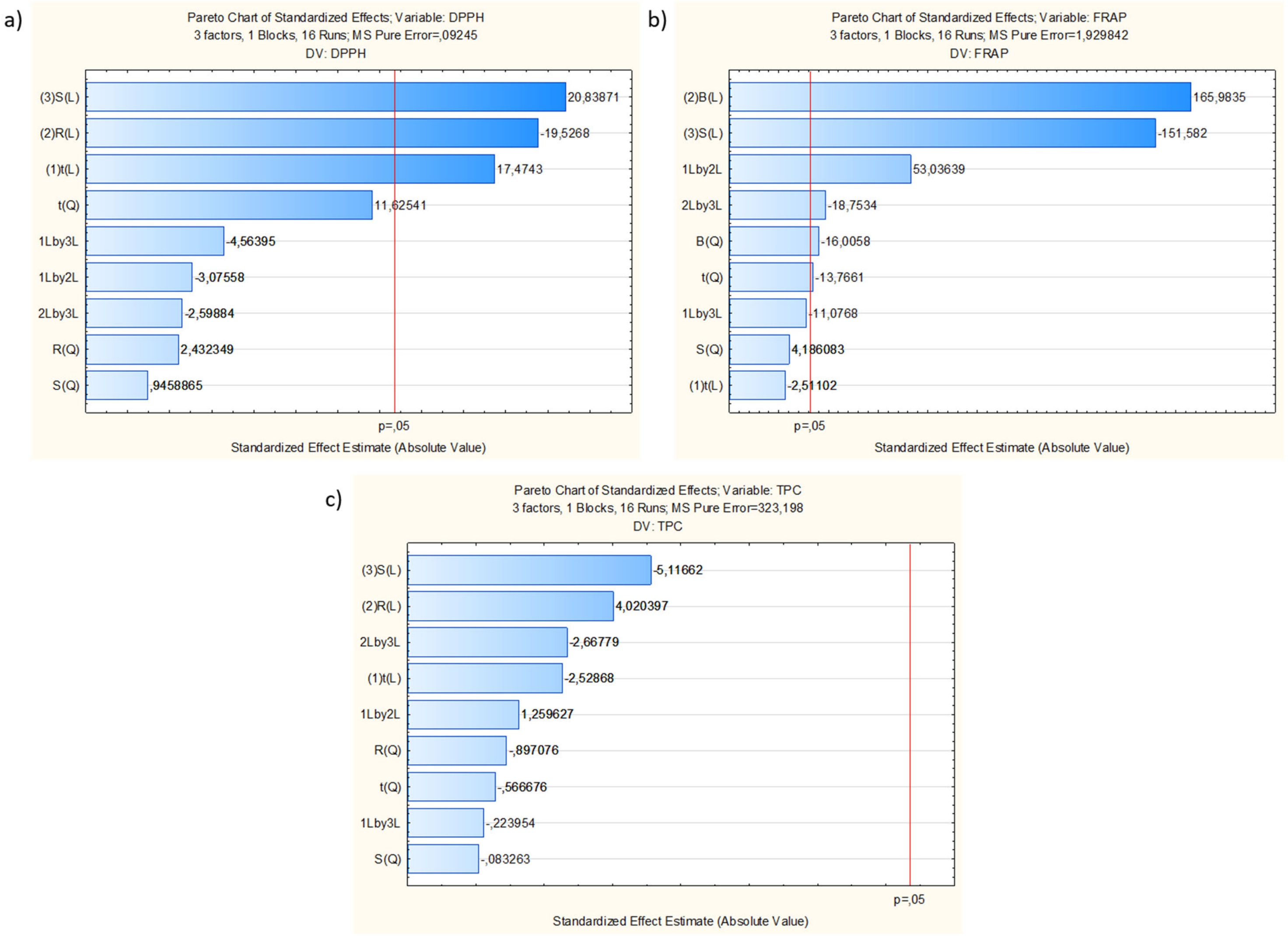

Figure S1. Pareto charts of the standardized effects of independent variables t, R and S in dependent variables DPPH (a), FRAP (b) and TPC (c) in the vortex-assisted extraction.

Table S5. Regression coefficients, standard error (SE) and *p*-value of the second-order polynomial models for optimization of the extraction of antioxidants from *F. spiralis*, using vortex-assisted extraction. t: extraction time (s); R: biomass:solvent ratio (g biomass/100 mL solvent); S: solvent (% of water in water:ethanol mixture); L: Linear effect; Q: Quadratic effect; 1Lby2L: interaction between the linear effect of t and the linear effect of R; 1Lby3L: interaction between the linear effect of t and the linear effect of S; 2Lby3L: interaction between the linear effect of R and the linear effect of S. Significant parameters are presented in red.

	DPPH						*FRAP*						*TPC*					
	Coeff.	SE	t(1)	p	-95,%	+95,%	Coeff.	SE	t(1)	p	-95,%	+95,%	Coeff.	SE	t(1)	p	-95,%	+95,%
Interc.	3,63	0,93	3,90	0,160	-8,18	15,44	235,29	4,25	55,39	0,011	181,31	289,26	62,51	54,97	1,14	0,459	-636,00	761,01
(1)t (L)	-0,02	0,01	-3,85	0,162	-0,10	0,05	-0,17	0,03	-5,92	0,107	-0,53	0,19	-0,13	0,37	-0,37	0,777	-4,78	4,51
t (Q)	0,00	0,00	11,63	0,055	0,00	0,00	0,00	0,00	-13,77	0,046	0,00	0,00	0,00	0,00	-0,57	0,672	-0,01	0,01
(2)R (L)	-0,09	0,03	-2,86	0,214	-0,51	0,32	3,57	0,15	23,74	0,027	1,66	5,48	3,57	1,95	1,83	0,318	-21,17	28,31
R (Q)	0,00	0,00	2,43	0,248	-0,01	0,01	-0,04	0,00	-16,01	0,040	-0,06	-0,01	-0,03	0,03	-0,90	0,535	-0,40	0,34
(3)S (L)	0,10	0,02	6,41	0,099	-0,10	0,29	-1,19	0,07	-17,06	0,037	-2,08	-0,30	0,36	0,90	0,39	0,761	-11,14	11,85
S (Q)	0,00	0,00	0,95	0,518	0,00	0,00	0,00	0,00	4,19	0,149	0,00	0,01	0,00	0,01	-0,08	0,947	-0,09	0,08
1L by 2L	0,00	0,00	-3,08	0,200	0,00	0,00	0,02	0,00	53,04	0,012	0,02	0,03	0,01	0,01	1,26	0,427	-0,06	0,08
1L by 3L	0,00	0,00	-4,56	0,137	0,00	0,00	0,00	0,00	-11,08	0,057	0,00	0,00	0,00	0,00	-0,22	0,860	-0,03	0,03
2L by 3L	0,00	0,00	-2,60	0,234	0,00	0,00	-0,02	0,00	-18,75	0,034	-0,04	-0,01	-0,04	0,01	-2,67	0,228	-0,23	0,15

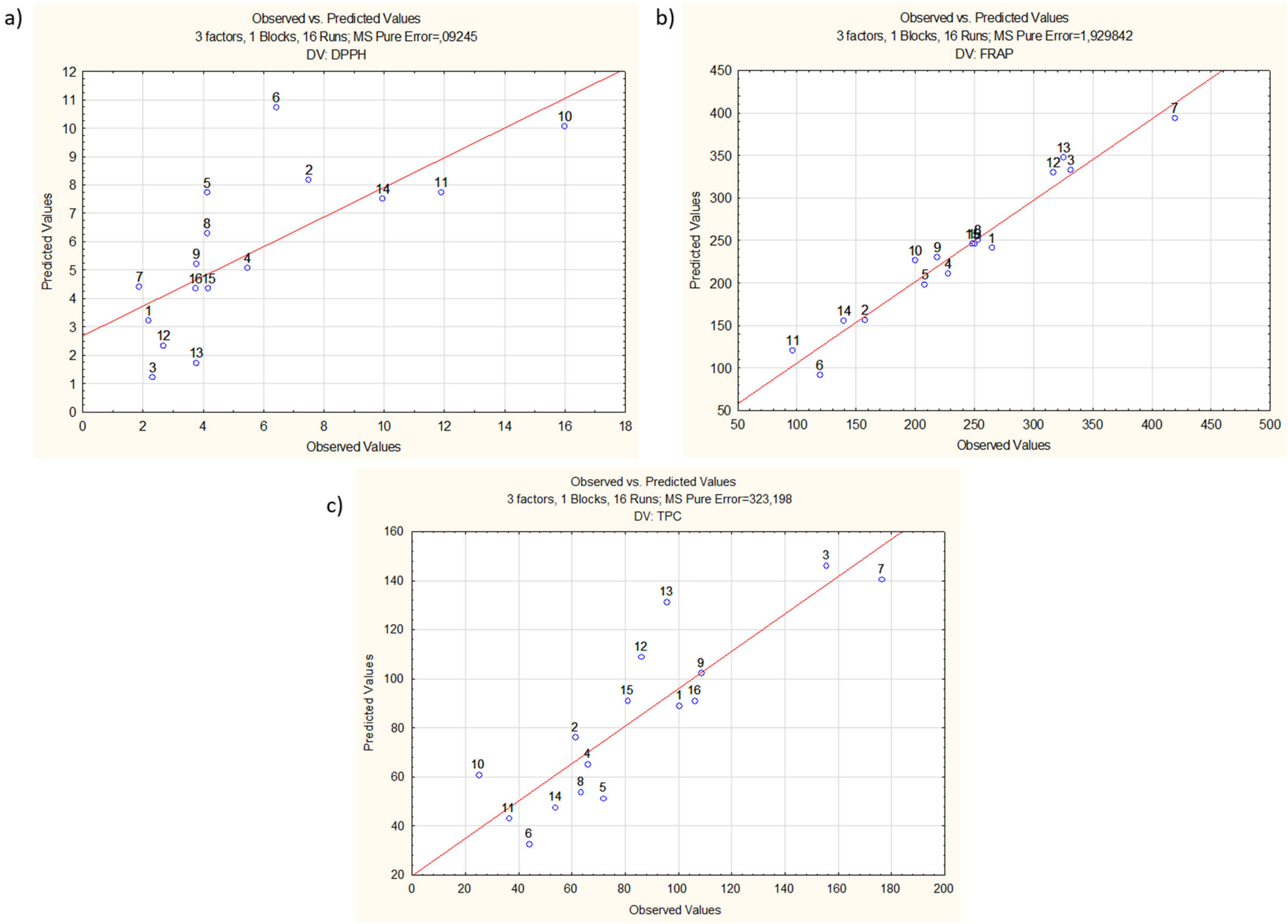

Figure S2. Observed vs. predicted DPPH (a), FRAP (b) and TPC (c) values for the vortex-assisted extraction.

Table S6. Observed, predicted, and residual values for the vortex-assisted extraction.

	DPPH			*FRAP*			*TPC*		
	Observed	Predictd	Resids	Observed	Predictd	Resids	Observed	Predictd	Resids
1	2,19	3,20	-1,02	265,06	241,31	23,75	100,46	89,13	11,33
2	7,51	8,17	-0,66	157,30	156,65	0,65	61,16	76,11	-14,95
3	2,33	1,21	1,12	331,37	332,43	-1,05	155,63	146,15	9,48
4	5,47	5,06	0,41	227,70	210,92	16,78	65,86	65,30	0,56
5	4,13	7,72	-3,59	208,01	198,21	9,80	71,70	51,36	20,34
6	6,43	10,73	-4,30	119,42	91,79	27,63	44,07	32,65	11,42
7	1,89	4,41	-2,52	419,45	393,52	25,93	176,26	140,41	35,85
8	4,13	6,30	-2,17	253,09	250,25	2,83	63,43	53,86	9,57
9	3,78	5,22	-1,44	218,84	229,88	-11,04	108,49	102,23	6,26
10	16,00	10,05	5,95	200,15	226,70	-26,55	25,04	60,85	-35,82
11	11,90	7,74	4,16	96,51	120,46	-23,95	36,48	43,13	-6,65
12	2,68	2,34	0,34	316,70	330,34	-13,64	86,01	108,91	-22,90
13	3,78	1,74	2,04	325,37	347,30	-21,93	95,77	131,48	-35,71
14	9,97	7,50	2,46	139,97	155,64	-15,67	53,91	47,76	6,15
15	4,18	4,35	-0,17	248,31	246,06	2,24	80,83	91,01	-10,18
16	3,75	4,35	-0,60	250,27	246,06	4,21	106,26	91,01	15,25

2.2. Ultra-Turrax® homogenizer

Table S7. Analysis of variance (ANOVA) of the second-order polynomial model for optimization of the extraction of antioxidants from *F. spiralis*, using Ultra-Turrax® homogenizer. t: extraction time (s); R: biomass:solvent ratio (g biomass/100 mL solvent); S: solvent (% of water in water:ethanol mixture); SS: Sum of squares; d.f.: Degree of freedom; MS: Mean square; F: F value; p: probability; L: Linear effect; Q: Quadratic effect; 1Lby2L: interaction between the linear effect of t and the linear effect of R; 1Lby3L: interaction between the linear effect of t and the linear effect of S; 2Lby3L: interaction between the linear effect of R and the linear effect of S. All parameters were non-significant.

	DPPH					*FRAP*					*TPC*				
	SS	df	MS	F	p	SS	df	MS	F	p	SS	df	MS	F	p
(1)t (L)	22,44	1	22,44	1,53	0,432	12481,50	1	12481,50	0,47	0,616	55,27	1	55,27	0,05	0,862
t (Q)	1,35	1	1,35	0,09	0,812	105683,40	1	105683,40	4,01	0,295	1,05	1	1,05	0,00	0,981
(2)R (L)	205,35	1	205,35	14,04	0,166	20892,90	1	20892,90	0,79	0,537	58,72	1	58,72	0,05	0,858
R (Q)	14,28	1	14,28	0,98	0,504	16,60	1	16,60	0,00	0,984	175,33	1	175,33	0,15	0,763
(3)S (L)	162,68	1	162,68	11,13	0,185	7503,30	1	7503,30	0,28	0,688	11120,16	1	11120,16	9,71	0,198
S (Q)	93,48	1	93,48	6,39	0,240	30683,20	1	30683,20	1,17	0,476	5239,28	1	5239,28	4,58	0,278
1L by 2L	8,63	1	8,63	0,59	0,583	5966,30	1	5966,30	0,23	0,717	75,84	1	75,84	0,07	0,840
1L by 3L	32,68	1	32,68	2,24	0,375	34358,80	1	34358,80	1,30	0,458	30,41	1	30,41	0,03	0,897
2L by 3L	85,96	1	85,96	5,88	0,249	50368,90	1	50368,90	1,91	0,399	1786,30	1	1786,30	1,56	0,430
Lack of Fit	55,54	5	11,11	0,76	0,697	279255,20	5	55851,00	2,12	0,477	2111,52	5	422,30	0,37	0,839
Pure Error	14,62	1	14,62			26336,90	1	26336,90			1145,16	1	1145,16		
Total SS	692,11	15				572310,00	15				22773,02	15			
R^2			0.899					0.466					0.857		
R^2 adj.			0.747					0.000					0.642		

Table S8. Effect estimates of variables in the extraction with Ultra-Turrax® homogenizer. t: extraction time (s); R: biomass:solvent ratio (g biomass/100 mL solvent); S: solvent (% of water in water:ethanol mixture); L: Linear effect; Q: Quadratic effect; 1Lby2L: interaction between the linear effect of t and the linear effect of R; 1Lby3L: interaction between the linear effect of t and the linear effect of S; 2Lby3L: interaction between the linear effect of R and the linear effect of S. Significant parameters are presented in red.

	Parameter	Effect	Std.Err. - Pure Err	t(1)	p	-95,% - Cnf.Limt	+95,% - Cnf.Limt	Coeff.	Std.Err. - Coeff.	-95,% - Cnf.Limt	+95,% - Cnf.Limt
DPPH	Mean/Interc.	18,68	2,70	6,93	0,091	-15,57	52,94	18,68	2,70	-15,57	52,94
	(1)t (L)	-2,56	2,07	-1,24	0,432	-28,86	23,73	-1,28	1,03	-14,43	11,87
	t (Q)	-0,76	2,51	-0,30	0,812	-32,69	31,16	-0,38	1,26	-16,34	15,58
	(2)R (L)	-7,76	2,07	-3,75	0,166	-34,05	18,54	-3,88	1,03	-17,02	9,27
	R (Q)	-2,48	2,51	-0,99	0,504	-34,41	29,44	-1,24	1,26	-17,20	14,72
	(3)S (L)	6,90	2,07	3,34	0,185	-19,39	33,20	3,45	1,03	-9,70	16,60
	S (Q)	-6,35	2,51	-2,53	0,240	-38,28	25,57	-3,18	1,26	-19,14	12,79
	1L by 2L	-2,08	2,70	-0,77	0,583	-36,43	32,28	-1,04	1,35	-18,22	16,14
	1L by 3L	4,04	2,70	1,50	0,375	-30,31	38,40	2,02	1,35	-15,16	19,20
	2L by 3L	-6,56	2,70	-2,42	0,249	-40,91	27,80	-3,28	1,35	-20,46	13,90
FRAP	Mean/Interc.	686,38	114,42	6,00	0,105	-767,43	2140,19	686,38	114,42	-767,43	2140,19
	(1)t (L)	-60,46	87,83	-0,69	0,616	-1176,43	1055,51	-30,23	43,91	-588,22	527,75
	t (Q)	213,62	106,64	2,00	0,295	-1141,34	1568,57	106,81	53,32	-570,67	784,29
	(2)R (L)	78,23	87,83	0,89	0,537	-1037,74	1194,20	39,11	43,91	-518,87	597,10
	R (Q)	2,68	106,64	0,03	0,984	-1352,28	1357,64	1,34	53,32	-676,14	678,82
	(3)S (L)	-46,88	87,83	-0,53	0,688	-1162,85	1069,09	-23,44	43,91	-581,43	534,55
	S (Q)	115,10	106,64	1,08	0,476	-1239,86	1470,06	57,55	53,32	-619,93	735,03
	1L by 2L	54,62	114,75	0,48	0,717	-1403,47	1512,70	27,31	57,38	-701,73	756,35
	1L by 3L	-131,07	114,75	-1,14	0,458	-1589,16	1327,02	-65,54	57,38	-794,58	663,51
	2L by 3L	158,70	114,75	1,38	0,399	-1299,39	1616,78	79,35	57,38	-649,69	808,39
TPC	*Mean/Interc.*	124,32	23,86	5,21	0,121	-178,83	427,47	124,32	23,86	-178,83	427,47
	(1)t (L)	4,02	18,31	0,22	0,862	-228,68	236,73	2,01	9,16	-114,34	118,36
	t (Q)	0,67	22,24	0,03	0,981	-281,87	283,21	0,34	11,12	-140,93	141,61
	(2)R (L)	4,15	18,31	0,23	0,858	-228,56	236,85	2,07	9,16	-114,28	118,43
	R (Q)	8,70	22,24	0,39	0,763	-273,84	291,24	4,35	11,12	-136,92	145,62
	(3)S (L)	-57,07	18,31	-3,12	0,198	-289,77	175,63	-28,54	9,16	-144,89	87,82
	S (Q)	47,56	22,24	2,14	0,278	-234,98	330,10	23,78	11,12	-117,49	165,05
	1L by 2L	6,16	23,93	0,26	0,840	-297,88	310,20	3,08	11,96	-148,94	155,10
	1L by 3L	-3,90	23,93	-0,16	0,897	-307,94	300,14	-1,95	11,96	-153,97	150,07
	2L by 3L	29,89	23,93	1,25	0,430	-274,16	333,93	14,94	11,96	-137,08	166,96

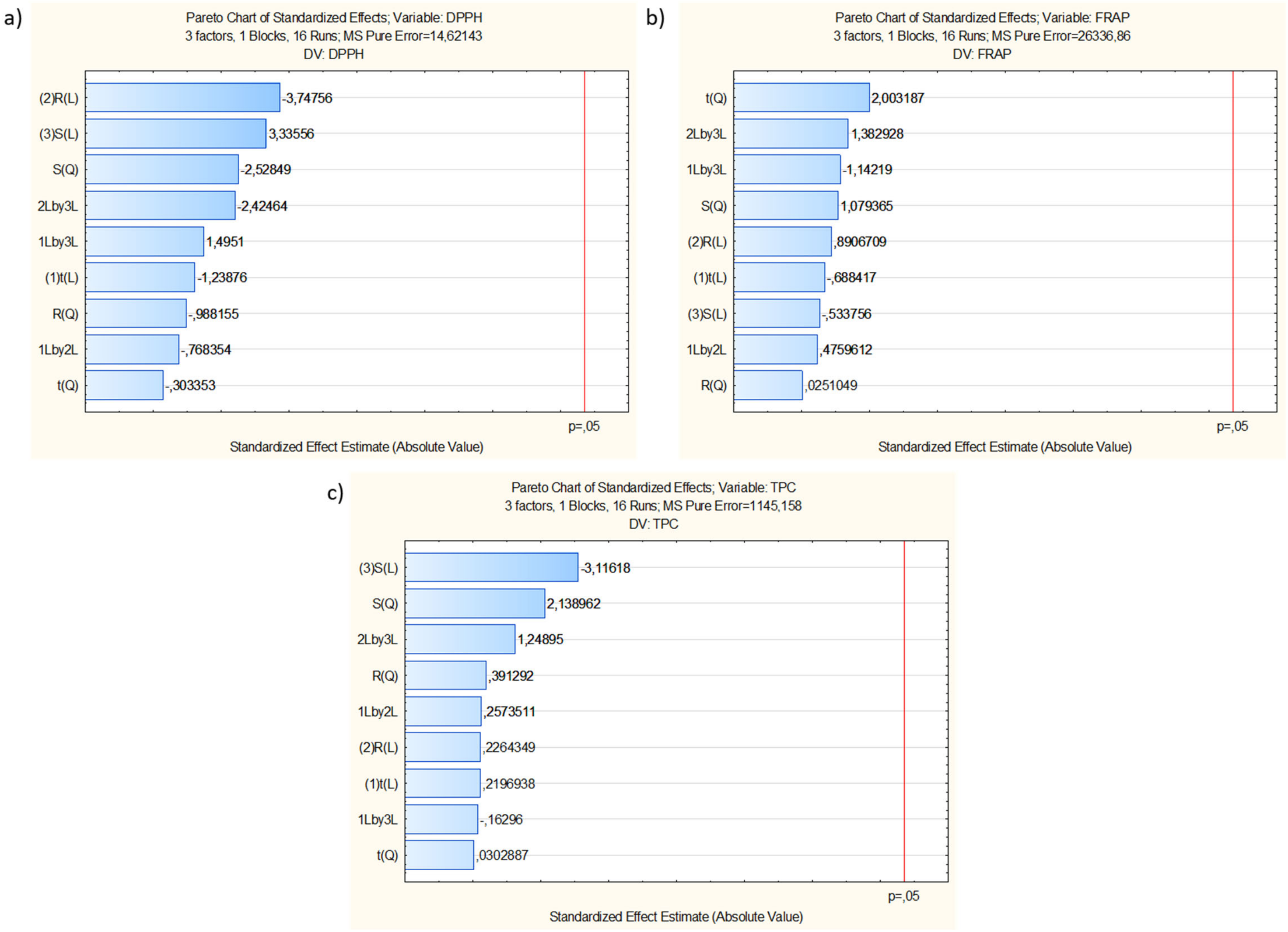

Figure S3. Pareto charts of the standardized effects of independent variables t, R and S in dependent variables DPPH (a), FRAP (b) and TPC (c) in the extraction of antioxidants with Ultra-Turrax® homogenizer.

Table S9. Regression coefficients, standard error (SE) and *p*-value of the second-order polynomial models for optimization of the extraction of antioxidants from *F. spiralis*, using Ultra-Turrax® homogenizer. t: extraction time (s); R: biomass:solvent ratio (g biomass/100 mL solvent); S: solvent (% of water in water:ethanol mixture); L: Linear effect; Q: Quadratic effect; 1Lby2L: interaction between the linear effect of t and the linear effect of R; 1Lby3L: interaction between the linear effect of t and the linear effect of S; 2Lby3L: interaction between the linear effect of R and the linear effect of S. Significant parameters are presented in red.

	DPPH						*FRAP*						*TPC*					
	Coeff.	SE	t(1)	p	-95,%	+95,%	Coeff.	SE	t(1)	p	-95,%	+95,%	Coeff.	SE	t(1)	p	-95,%	+95,%
Interc.	0,91	11,69	0,08	0,950	-147,66	149,48	1454,03	496,25	2,93	0,209	-4851,40	7759,46	298,10	103,48	2,88	0,213	-1016,72	1612,92
(1)t (L)	-0,02	0,08	-0,20	0,877	-1,00	0,97	-5,09	3,30	-1,54	0,366	-47,04	36,85	-0,02	0,69	-0,03	0,980	-8,77	8,73
t (Q)	0,00	0,00	-0,30	0,812	0,00	0,00	0,02	0,01	2,00	0,295	-0,09	0,12	0,00	0,00	0,03	0,981	-0,02	0,02
(2)R (L)	0,58	0,41	1,40	0,394	-4,68	5,84	-10,89	17,57	-0,62	0,647	-234,19	212,42	-3,17	3,66	-0,87	0,546	-49,73	43,39
R (Q)	-0,01	0,01	-0,99	0,504	-0,08	0,07	0,01	0,26	0,03	0,984	-3,32	3,33	0,02	0,05	0,39	0,763	-0,67	0,72
(3)S (L)	0,54	0,19	2,79	0,219	-1,91	2,98	-7,63	8,17	-0,93	0,522	-111,38	96,12	-4,43	1,70	-2,60	0,234	-26,07	17,20
S (Q)	0,00	0,00	-2,53	0,240	-0,02	0,01	0,07	0,06	1,08	0,476	-0,70	0,83	0,03	0,01	2,14	0,278	-0,13	0,19
1L by 2L	0,00	0,00	-0,77	0,583	-0,02	0,01	0,02	0,05	0,48	0,717	-0,61	0,66	0,00	0,01	0,26	0,840	-0,13	0,14
1L by 3L	0,00	0,00	1,50	0,375	-0,01	0,01	-0,03	0,02	-1,14	0,458	-0,33	0,28	0,00	0,01	-0,16	0,897	-0,06	0,06
2L by 3L	-0,01	0,00	-2,42	0,249	-0,05	0,03	0,19	0,14	1,38	0,399	-1,53	1,91	0,04	0,03	1,25	0,430	-0,32	0,39

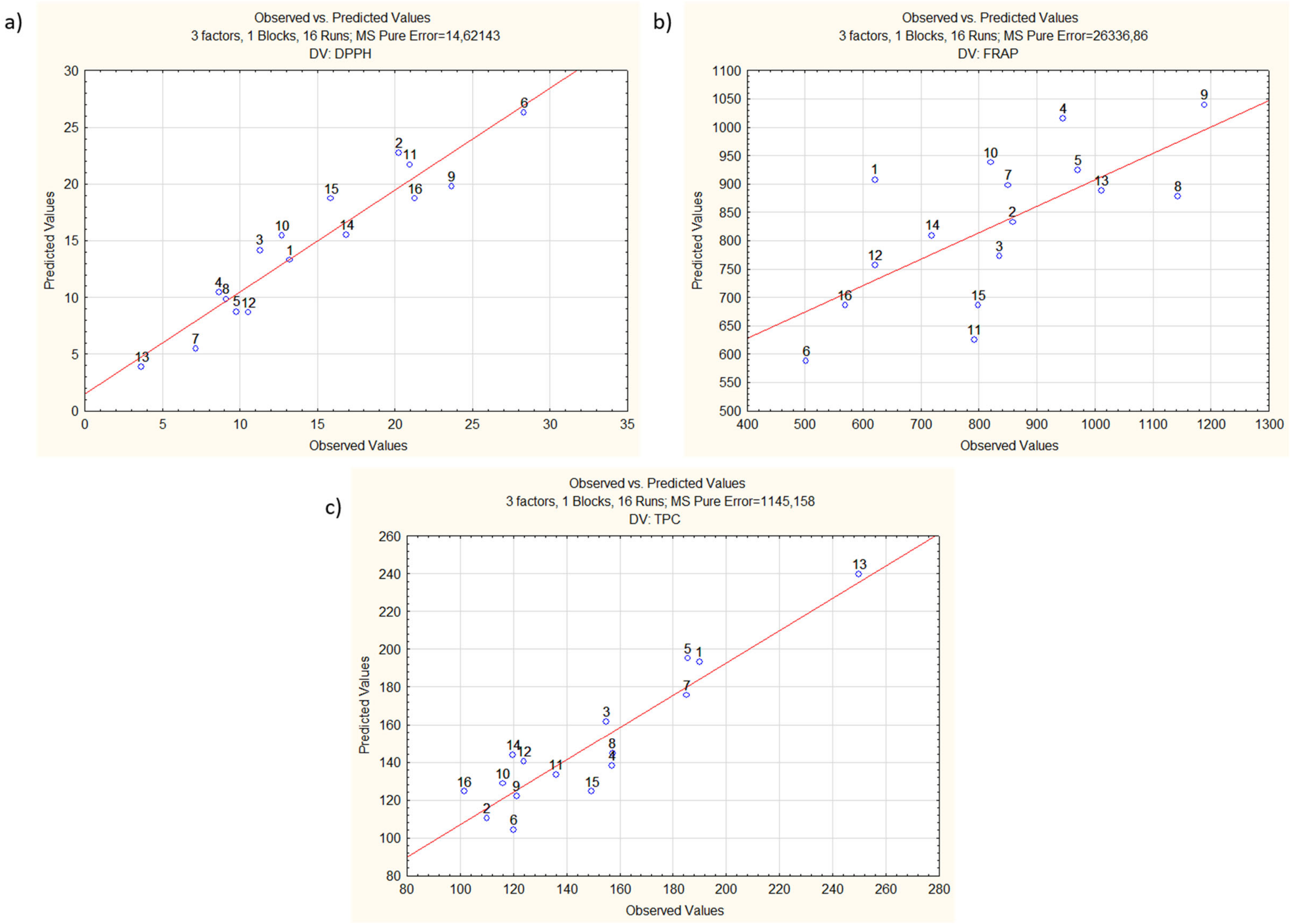

Figure S4. Observed vs. predicted DPPH (a), FRAP (b) and TPC (c) values for the extraction with Ultra-Turrax® homogenizer.

Table S10. Observed, predicted, and residual values for the extraction with Ultra-Turrax® homogenizer.

	DPPH			*FRAP*			*TPC*		
	Observed	Predictd	Resids	Observed	Predictd	Resids	Observed	Predictd	Resids
1	13,25	13,29	-0,05	620,74	907,76	-287,02	189,90	193,31	-3,41
2	20,24	22,71	-2,47	858,11	833,25	24,86	110,05	110,25	-0,21
3	11,27	14,17	-2,90	835,03	772,67	62,36	154,89	161,41	-6,52
4	8,64	10,48	-1,83	945,63	1015,56	-69,92	157,02	138,13	18,89
5	9,77	8,77	1,01	971,09	923,75	47,35	185,60	195,07	-9,48
6	28,34	26,27	2,07	502,16	587,10	-84,94	120,16	104,22	15,94
7	7,14	5,49	1,65	850,46	897,90	-47,44	185,11	175,49	9,62
8	9,10	9,88	-0,78	1143,08	878,64	264,44	157,23	144,41	12,82
9	23,67	19,76	3,91	1188,81	1039,32	149,49	121,22	121,89	-0,67
10	12,70	15,45	-2,75	820,07	937,63	-117,56	116,01	128,65	-12,65
11	20,96	21,69	-0,73	791,74	624,39	167,35	135,98	133,14	2,84
12	10,55	8,65	1,90	620,53	755,95	-135,42	123,95	140,11	-16,16
13	3,67	3,89	-0,23	1011,33	888,58	122,75	249,93	239,57	10,36
14	16,89	15,50	1,39	718,92	809,74	-90,82	119,92	143,59	-23,67
15	15,88	18,68	-2,80	798,40	686,38	112,01	149,39	124,32	25,07
16	21,28	18,68	2,60	568,89	686,38	-117,49	101,53	124,32	-22,79

2.3. Ultrasonic bath

Table S11. Analysis of variance (ANOVA) of the second-order polynomial model for optimization of the extraction of antioxidants from *F. spiralis*, using ultrasonic bath-assisted extraction. t: extraction time (s); R: biomass:solvent ratio (g biomass/100 mL solvent); S: solvent (% of water in water:ethanol mixture); SS: Sum of squares; df.: Degree of freedom; MS: Mean square; F: F value; p: probability; L: Linear effect; Q: Quadratic effect; 1Lby2L: interaction between the linear effect of t and the linear effect of R; 1Lby3L: interaction between the linear effect of t and the linear effect of S; 2Lby3L: interaction between the linear effect of R and the linear effect of S. Significant parameters are presented in red.

	DPPH					*FRAP*					*TPC*				
	SS	df	MS	F	p	SS	df	MS	F	p	SS	df	MS	F	p
(1)t (L)	245,43	1	245,43	31,59	0,112	12,20	1	12,21	6,31	0,241	1916,50	1	1916,54	232487,00	0,001
t (Q)	159,25	1	159,25	20,50	0,138	253,50	1	253,51	130,90	0,056	12926,20	1	12926,18	1568021,00	0,001
(2)R (L)	574,16	1	574,16	73,90	0,074	53357,80	1	53357,78	27550,51	0,004	20978,90	1	20978,91	2544865,00	0,000
R (Q)	57,30	1	57,30	7,37	0,225	362,50	1	362,48	187,16	0,046	10,30	1	10,34	1254,00	0,018
(3)S (L)	4079,67	1	4079,67	525,08	0,028	39681,90	1	39681,93	20489,18	0,004	12879,00	1	12879,04	1562303,00	0,001
S (Q)	1305,39	1	1305,39	168,01	0,049	23,30	1	23,32	12,04	0,179	1576,90	1	1576,93	191291,00	0,001
1L by 2L	3,66	1	3,66	0,47	0,617	5447,70	1	5447,73	2812,86	0,012	133,90	1	133,90	16243,00	0,005
1L by 3L	28,98	1	28,98	3,73	0,304	237,60	1	237,63	122,70	0,057	8333,50	1	8333,54	1010908,00	0,001
2L by 3L	1083,20	1	1083,20	139,41	0,054	681,10	1	681,12	351,69	0,034	6124,70	1	6124,73	742966,00	0,001
Lack of Fit	2803,10	5	560,62	72,15	0,089	6133,30	5	1226,66	633,37	0,030	46009,50	5	9201,90	1116245,00	0,001
Pure Error	7,77	1	7,77			1,90	1	1,94			0,00	1	0,01		
Total SS	10785,11	15				106032,80	15				119089,70	15			
R^2			0.739					0.942					0.614		
R^2 *adj.*			0.348					0.855					0.034		

Table S12. Effect estimates of variables in ultrasonic bath-assisted extraction. t: extraction time (s); R: biomass:solvent ratio (g biomass/100 mL solvent); S: solvent (% of water in water:ethanol mixture); L: Linear effect; Q: Quadratic effect; 1Lby2L: interaction between the linear effect of t and the linear effect of R; 1Lby3L: interaction between the linear effect of t and the linear effect of S; 2Lby3L: interaction between the linear effect of R and the linear effect of S. Significant parameters are presented in red.

	Parameters	Effect	Std.Err. - Pure Err	t(1)	p	-95,% - Cnf.Limt	+95,% - Cnf.Limt	Coeff.	Std.Err. - Coeff.	-95,% - Cnf.Limt	+95,% - Cnf.Limt
DPPH	Mean/Interc.	9,79	1,97	4,98	0,126	-15,18	34,76	9,79	1,97	-15,18	34,76
	(1)t (L)	-8,48	1,51	-5,62	0,112	-27,65	10,69	-4,24	0,75	-13,82	5,34
	t (Q)	8,29	1,83	4,53	0,138	-14,98	31,56	4,15	0,92	-7,49	15,78
	(2)R (L)	-12,97	1,51	-8,60	0,074	-32,14	6,20	-6,48	0,75	-16,07	3,10
	R (Q)	-4,97	1,83	-2,72	0,225	-28,25	18,30	-2,49	0,92	-14,12	9,15
	(3)S (L)	34,57	1,51	22,91	0,028	15,40	53,74	17,28	0,75	7,70	26,87
	S (Q)	23,74	1,83	12,96	0,049	0,47	47,01	11,87	0,92	0,23	23,51
	1L by 2L	1,35	1,97	0,69	0,617	-23,69	26,40	0,68	0,99	-11,85	13,20
	1L by 3L	-3,81	1,97	-1,93	0,304	-28,85	21,24	-1,90	0,99	-14,43	10,62
	2L by 3L	-23,27	1,97	-11,81	0,054	-48,32	1,77	-11,64	0,99	-24,16	0,89
FRAP	Mean/Interc.	245,63	0,98	250,34	0,003	233,16	258,09	245,63	0,98	233,16	258,09
	(1)t (L)	-1,89	0,75	-2,51	0,241	-11,46	7,68	-0,95	0,38	-5,73	3,84
	t (Q)	-10,46	0,91	-11,44	0,056	-22,08	1,16	-5,23	0,46	-11,04	0,58
	(2)R (L)	125,01	0,75	165,98	0,004	115,44	134,58	62,51	0,38	57,72	67,29
	R (Q)	-12,51	0,91	-13,68	0,046	-24,13	-0,89	-6,26	0,46	-12,06	-0,45
	(3)S (L)	-107,81	0,75	-143,14	0,004	-117,38	-98,24	-53,90	0,38	-58,69	-49,12
	S (Q)	-3,17	0,91	-3,47	0,179	-14,79	8,45	-1,59	0,46	-7,40	4,22
	1L by 2L	52,19	0,98	53,04	0,012	39,69	64,69	26,10	0,49	19,84	32,35
	1L by 3L	-10,90	0,98	-11,08	0,057	-23,40	1,60	-5,45	0,49	-11,70	0,80
	2L by 3L	-18,45	0,98	-18,75	0,034	-30,96	-5,95	-9,23	0,49	-15,48	-2,98
TPC	Mean/Interc.	167,38	0,06	2614,81	<0,001	166,57	168,20	167,38	0,06	166,57	168,20
	(1)t (L)	23,69	0,05	482,17	0,001	23,07	24,32	11,85	0,02	11,53	12,16
	t (Q)	74,71	0,06	1252,21	0,001	73,95	75,47	37,35	0,03	36,97	37,73
	(2)R (L)	78,39	0,05	1595,26	<0,001	77,76	79,01	39,19	0,02	38,88	39,51
	R (Q)	2,11	0,06	35,41	0,018	1,35	2,87	1,06	0,03	0,68	1,44
	(3)S (L)	-61,42	0,05	-1249,92	0,001	-62,04	-60,79	-30,71	0,02	-31,02	-30,40
	S (Q)	-26,09	0,06	-437,37	0,001	-26,85	-25,34	-13,05	0,03	-13,43	-12,67
	1L by 2L	-8,18	0,06	-127,45	0,005	-9,00	-7,37	-4,09	0,03	-4,50	-3,68
	1L by 3L	64,55	0,06	1005,44	0,001	63,73	65,37	32,28	0,03	31,87	32,68
	2L by 3L	55,34	0,06	861,95	0,001	54,52	56,15	27,67	0,03	27,26	28,08

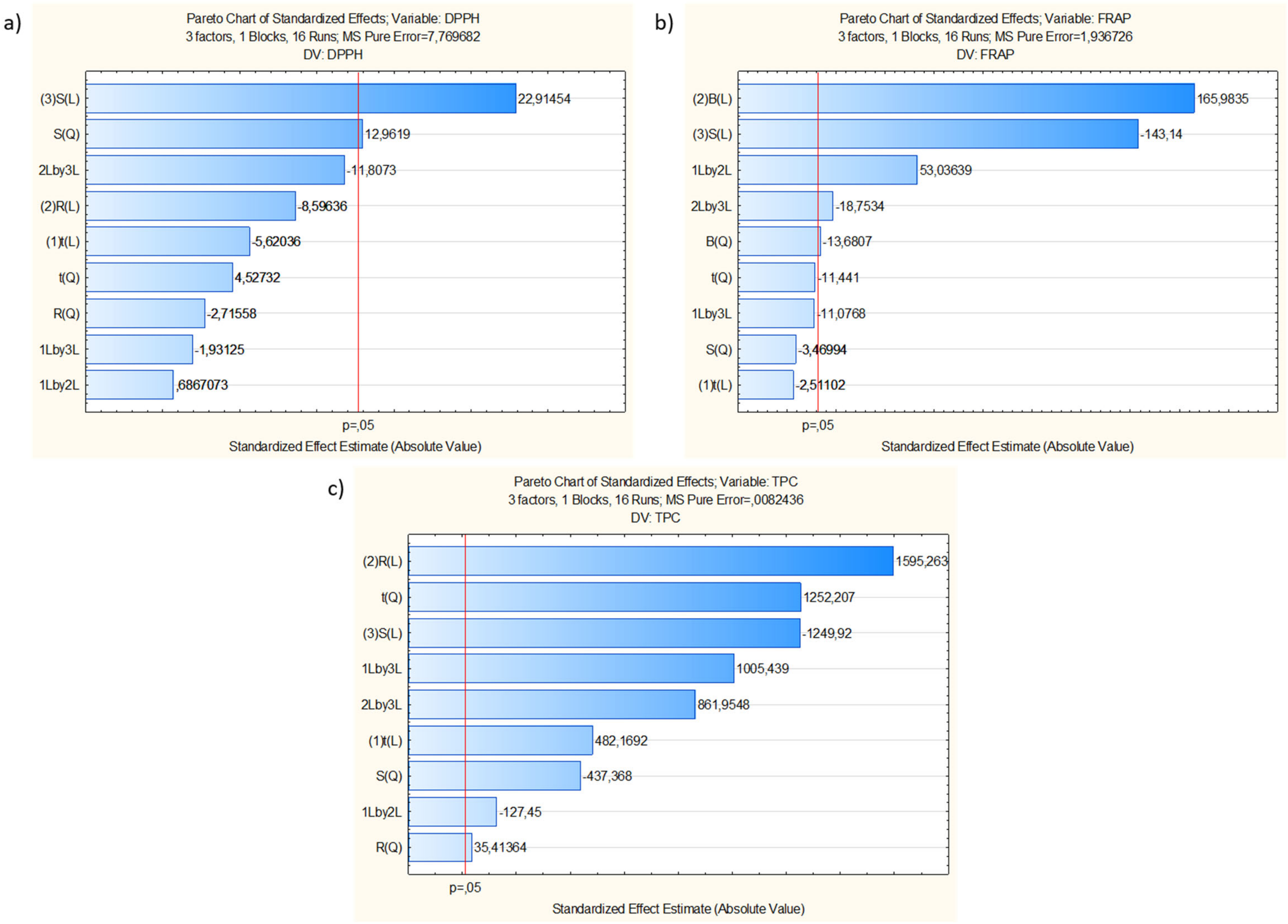

Figure S5. Pareto charts of the standardized effects of independent variables t, R and S in dependent variables DPPH (a), FRAP (b) and TPC (c) in the ultrasonic bath-assisted extraction.

Table S13. Regression coefficients, standard error (SE) and *p*-value of the second-order polynomial models for optimization of the extraction of antioxidants from *F. spiralis*, using ultrasonic bath-assisted extraction. t: extraction time (s); R: biomass:solvent ratio (g biomass/100 mL solvent); S: solvent (% of water in water:ethanol mixture); L: Linear effect; Q: Quadratic effect; 1Lby2L: interaction between the linear effect of t and the linear effect of R; 1Lby3L: interaction between the linear effect of t and the linear effect of S; 2Lby3L: interaction between the linear effect of R and the linear effect of S. Significant parameters are presented in red.

	DPPH						*FRAP*						*TPC*					
	Coeff.	SE	t(1)	p	-95,%	+95,%	Coeff.	SE	t(1)	p	-95,%	+95,%	Coeff.	SE	t(1)	p	-95,%	+95,%
Interc.	4,39	8,52	0,51	0,697	-103,91	112,69	227,60	4,26	53,48	0,012	173,53	281,67	428,73	0,28	1544,23	<0,001	425,21	432,26
(1)t (L)	-0,24	0,06	-4,24	0,147	-0,96	0,48	-0,22	0,03	-7,84	0,081	-0,58	0,14	-2,35	0,00	-1271,80	0,001	-2,37	-2,33
t (Q)	0,00	0,00	4,53	0,138	0,00	0,00	0,00	0,00	-11,44	0,056	0,00	0,00	0,01	0,00	1252,21	0,001	0,01	0,01
(2)R (L)	1,45	0,30	4,82	0,130	-2,38	5,29	3,31	0,15	21,94	0,029	1,39	5,22	-0,19	0,01	-19,81	0,032	-0,32	-0,07
R (Q)	-0,01	0,00	-2,72	0,225	-0,07	0,04	-0,03	0,00	-13,68	0,046	-0,06	0,00	0,01	0,00	35,41	0,018	0,00	0,01
(3)S (L)	0,08	0,14	0,59	0,660	-1,70	1,87	-0,69	0,07	-9,87	0,064	-1,58	0,20	-3,48	0,00	-762,66	0,001	-3,54	-3,43
S (Q)	0,01	0,00	12,96	0,049	0,00	0,03	0,00	0,00	-3,47	0,179	-0,01	0,00	-0,01	0,00	-437,37	0,001	-0,02	-0,01
1L by 2L	0,00	0,00	0,69	0,617	-0,01	0,01	0,02	0,00	53,04	0,012	0,02	0,03	0,00	0,00	-127,45	0,005	0,00	0,00
1L by 3L	0,00	0,00	-1,93	0,304	-0,01	0,00	0,00	0,00	-11,08	0,057	0,00	0,00	0,01	0,00	1005,44	0,001	0,01	0,01
2L by 3L	-0,03	0,00	-11,81	0,054	-0,06	0,00	-0,02	0,00	-18,75	0,034	-0,04	-0,01	0,07	0,00	861,95	0,001	0,06	0,07

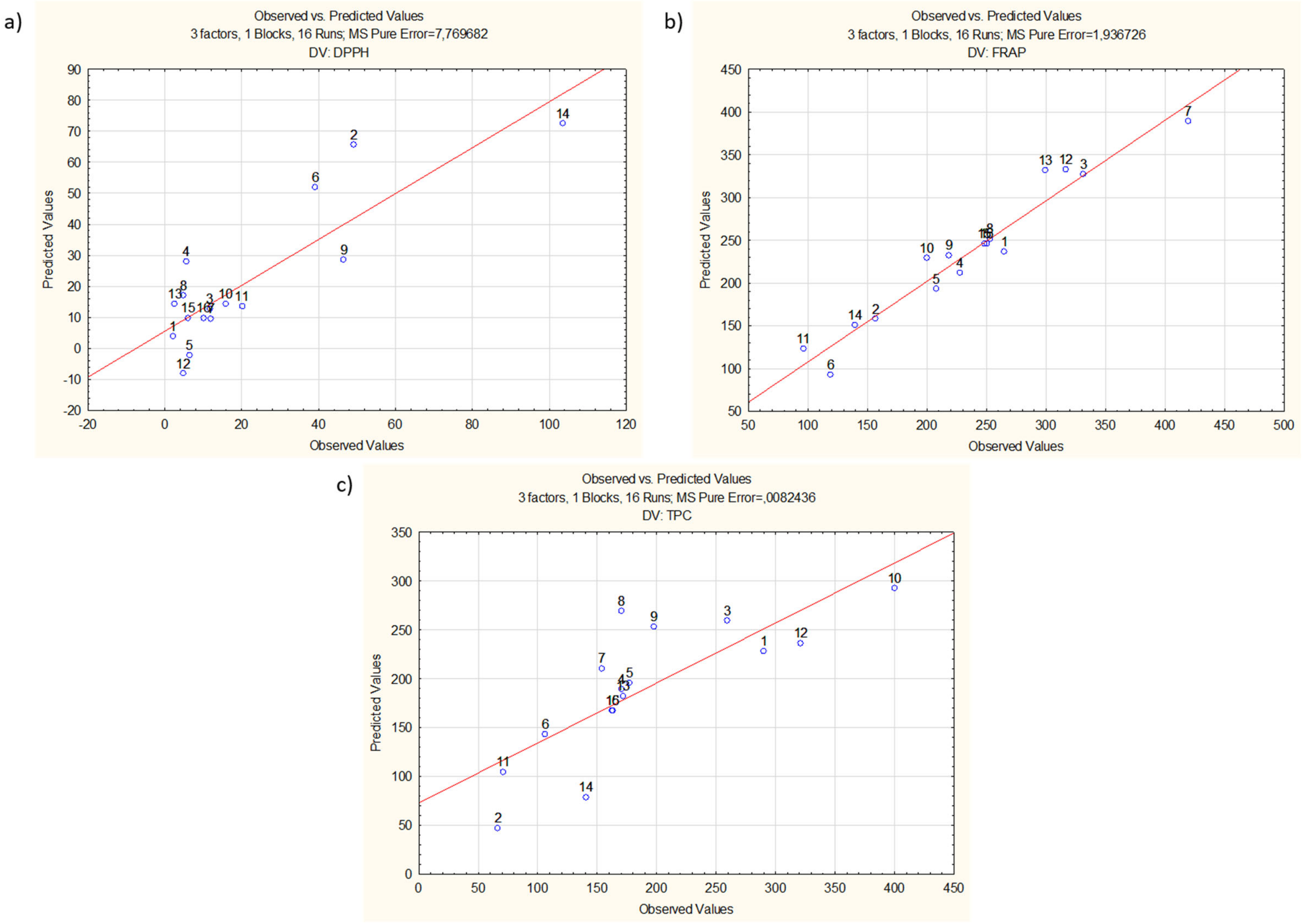

Figure S6. Observed vs. predicted DPPH (a), FRAP (b), and TPC (c) values for the ultrasonic bath-assisted extraction.

Table S14. Observed, predicted, and residual values for the ultrasonic bath-assisted extraction.

	DPPH			*FRAP*			*TPC*		
	Observed	Predictd	Resids	Observed	Predictd	Resids	Observed	Predictd	Resids
1	2,25	3,90	-1,65	265,10	236,31	28,78	290,25	228,27	61,98
2	49,25	65,54	-16,29	157,15	157,86	-0,71	66,72	46,96	19,76
3	11,81	12,85	-1,04	331,53	327,59	3,94	259,84	259,50	0,34
4	5,59	27,95	-22,36	227,67	212,23	15,44	170,66	188,87	-18,21
5	6,47	-2,13	8,60	207,95	193,13	14,82	177,32	195,59	-18,28
6	39,19	51,91	-12,72	119,20	92,88	26,32	106,57	143,39	-36,82
7	12,06	9,53	2,53	419,76	388,79	30,97	154,22	210,46	-56,24
8	4,91	17,02	-12,11	253,10	251,63	1,48	170,47	268,93	-98,46
9	46,60	28,65	17,95	218,79	232,42	-13,63	197,55	253,11	-55,56
10	15,90	14,39	1,51	200,08	229,24	-29,16	400,12	292,96	107,16
11	20,15	13,66	6,48	96,25	122,81	-26,56	71,03	104,45	-33,43
12	4,83	-8,15	12,98	316,83	333,06	-16,23	321,31	236,29	85,02
13	2,65	14,30	-11,65	299,70	331,79	-32,09	171,79	182,13	-10,33
14	103,55	72,43	31,12	139,79	150,48	-10,70	140,76	78,83	61,93
15	6,15	9,79	-3,64	248,31	245,63	2,69	162,89	167,38	-4,49
16	10,09	9,79	0,30	250,28	245,63	4,66	163,02	167,38	-4,36

2.4. Ultrasonic probe

Table S15. Analysis of variance (ANOVA) of the second-order polynomial model for optimization of the extraction of antioxidants from *F. spiralis*, using ultrasonic probe-assisted extraction. t: extraction time (s); R: biomass:solvent ratio (g biomass/100 mL solvent); S: solvent (% of water in water:ethanol mixture); SS: Sum of squares; d.f.: Degree of freedom; MS: Mean square; F: F value; p: probability; L: Linear effect; Q: Quadratic effect. All parameters were non-significant.

	DPPH					FRAP					TPC				
	SS	df	MS	F	p	SS	df	MS	F	p	SS	df	MS	F	p
(1)t (L)	1,46	1	1,46	0,27	0,694	6261,80	1	6261,75	0,40	0,642	2498,90	1	2498,94	0,06	0,850
t (Q)	2,60	1	2,60	0,48	0,613	4802,70	1	4802,70	0,31	0,679	10399,40	1	10399,40	0,24	0,710
(2)R (L)	110,70	1	110,70	20,64	0,138	11435,30	1	11435,28	0,73	0,550	36255,70	1	36255,74	0,83	0,529
R (Q)	123,48	1	123,48	23,03	0,131	13742,10	1	13742,13	0,87	0,521	9,60	1	9,59	0,00	0,991
(3)S (L)	334,15	1	334,15	62,31	0,080	96955,50	1	96955,52	6,17	0,244	25487,60	1	25487,60	0,59	0,584
S (Q)	2,86	1	2,86	0,53	0,599	34,80	1	34,83	0,00	0,970	2352,70	1	2352,75	0,05	0,854
1L by 2L	30,30	1	30,30	5,65	0,254	4368,80	1	4368,77	0,28	0,691	3579,40	1	3579,40	0,08	0,822
1L by 3L	11,67	1	11,67	2,18	0,379	656,70	1	656,70	0,04	0,872	3015,10	1	3015,11	0,07	0,836
2L by 3L	133,17	1	133,17	24,83	0,126	6968,80	1	6968,84	0,44	0,626	37276,90	1	37276,89	0,86	0,524
Lack of Fit	149,53	5	29,91	5,58	0,310	13797,20	5	2759,44	0,18	0,937	36421,70	5	7284,35	0,17	0,941
Pure Error	5,36	1	5,36			15719,90	1	15719,91			43424,00	1	43423,96		
Total SS	917,01	15				173636,00	15				200689,70	15			
R^2			0.831					0.830					0.602		
R^2 *adj.*			0.578					0.575					0.005		

Table S16. Effect estimates of variables in ultrasonic probe-assisted extraction. t: extraction time (s); R: biomass:solvent ratio (g biomass/100 mL solvent); S: solvent (% of water in water:ethanol mixture); L: Linear effect; Q: Quadratic effect; 1Lby2L: interaction between the linear effect of t and the linear effect of R; 1Lby3L: interaction between the linear effect of t and the linear effect of S; 2Lby3L: interaction between the linear effect of R and the linear effect of S. Significant parameters are presented in red.

	Parameters	Effect	Std.Err. - Pure Err	t(1)	p	-95,% - Cnf.Limt	+95,% - Cnf.Limt	Coeff.	Std.Err. - Coeff.	-95,% - Cnf.Limt	+95,% - Cnf.Limt
DPPH	Mean/Interc.	6,57	1,63	4,02	0,155	-14,17	27,32	6,57	1,63	-14,17	27,32
	(1)t (L)	-0,65	1,25	-0,52	0,694	-16,58	15,27	-0,33	0,63	-8,29	7,64
	t (Q)	1,06	1,52	0,70	0,613	-18,28	20,39	0,53	0,76	-9,14	10,20
	(2)R (L)	-5,69	1,25	-4,54	0,138	-21,62	10,23	-2,85	0,63	-10,81	5,12
	R (Q)	7,30	1,52	4,80	0,131	-12,03	26,64	3,65	0,76	-6,02	13,32
	(3)S (L)	9,89	1,25	7,89	0,080	-6,03	25,82	4,95	0,63	-3,02	12,91
	S (Q)	1,11	1,52	0,73	0,599	-18,22	20,45	0,56	0,76	-9,11	10,22
	1L by 2L	3,89	1,64	2,38	0,254	-16,91	24,70	1,95	0,82	-8,46	12,35
	1L by 3L	-2,42	1,64	-1,48	0,379	-23,22	18,39	-1,21	0,82	-11,61	9,20
	2L by 3L	-8,16	1,64	-4,98	0,126	-28,97	12,65	-4,08	0,82	-14,48	6,32
FRAP	Mean/Interc.	374,88	88,40	4,24	0,147	-748,31	1498,06	374,88	88,40	-748,31	1498,06
	(1)t (L)	42,83	67,85	0,63	0,642	-819,35	905,00	21,41	33,93	-409,68	452,50
	t (Q)	-45,54	82,39	-0,55	0,679	-1092,35	1001,28	-22,77	41,19	-546,18	500,64
	(2)R (L)	57,87	67,85	0,85	0,550	-804,30	920,05	28,94	33,93	-402,15	460,03
	R (Q)	-77,03	82,39	-0,93	0,521	-1123,84	969,78	-38,51	41,19	-561,92	484,89
	(3)S (L)	-168,52	67,85	-2,48	0,244	-1030,69	693,66	-84,26	33,93	-515,35	346,83
	S (Q)	-3,88	82,39	-0,05	0,970	-1050,69	1042,94	-1,94	41,19	-525,35	521,47
	1L by 2L	-46,74	88,66	-0,53	0,691	-1173,22	1079,75	-23,37	44,33	-586,61	539,87
	1L by 3L	-18,12	88,66	-0,20	0,872	-1144,61	1108,37	-9,06	44,33	-572,30	554,18
	2L by 3L	-59,03	88,66	-0,67	0,626	-1185,52	1067,46	-29,51	44,33	-592,76	533,73
TPC	Mean/Interc.	312,44	146,92	2,13	0,280	-1554,33	2179,21	312,44	146,92	-1554,33	2179,21
	(1)t (L)	-27,05	112,78	-0,24	0,850	-1460,02	1405,91	-13,53	56,39	-730,01	702,96
	t (Q)	-67,01	136,93	-0,49	0,710	-1806,85	1672,83	-33,50	68,46	-903,42	836,42
	(2)R (L)	103,05	112,78	0,91	0,529	-1329,92	1536,01	51,52	56,39	-664,96	768,01
	R (Q)	-2,03	136,93	-0,01	0,991	-1741,87	1737,80	-1,02	68,46	-870,94	868,90
	(3)S (L)	-86,40	112,78	-0,77	0,584	-1519,37	1346,56	-43,20	56,39	-759,68	673,28
	S (Q)	-31,87	136,93	-0,23	0,854	-1771,71	1707,97	-15,94	68,46	-885,86	853,98
	1L by 2L	-42,31	147,35	-0,29	0,822	-1914,56	1829,95	-21,15	73,67	-957,28	914,98
	1L by 3L	38,83	147,35	0,26	0,836	-1833,43	1911,09	19,41	73,67	-916,72	955,54
	2L by 3L	-136,52	147,35	-0,93	0,524	-2008,78	1735,74	-68,26	73,67	-1004,39	867,87

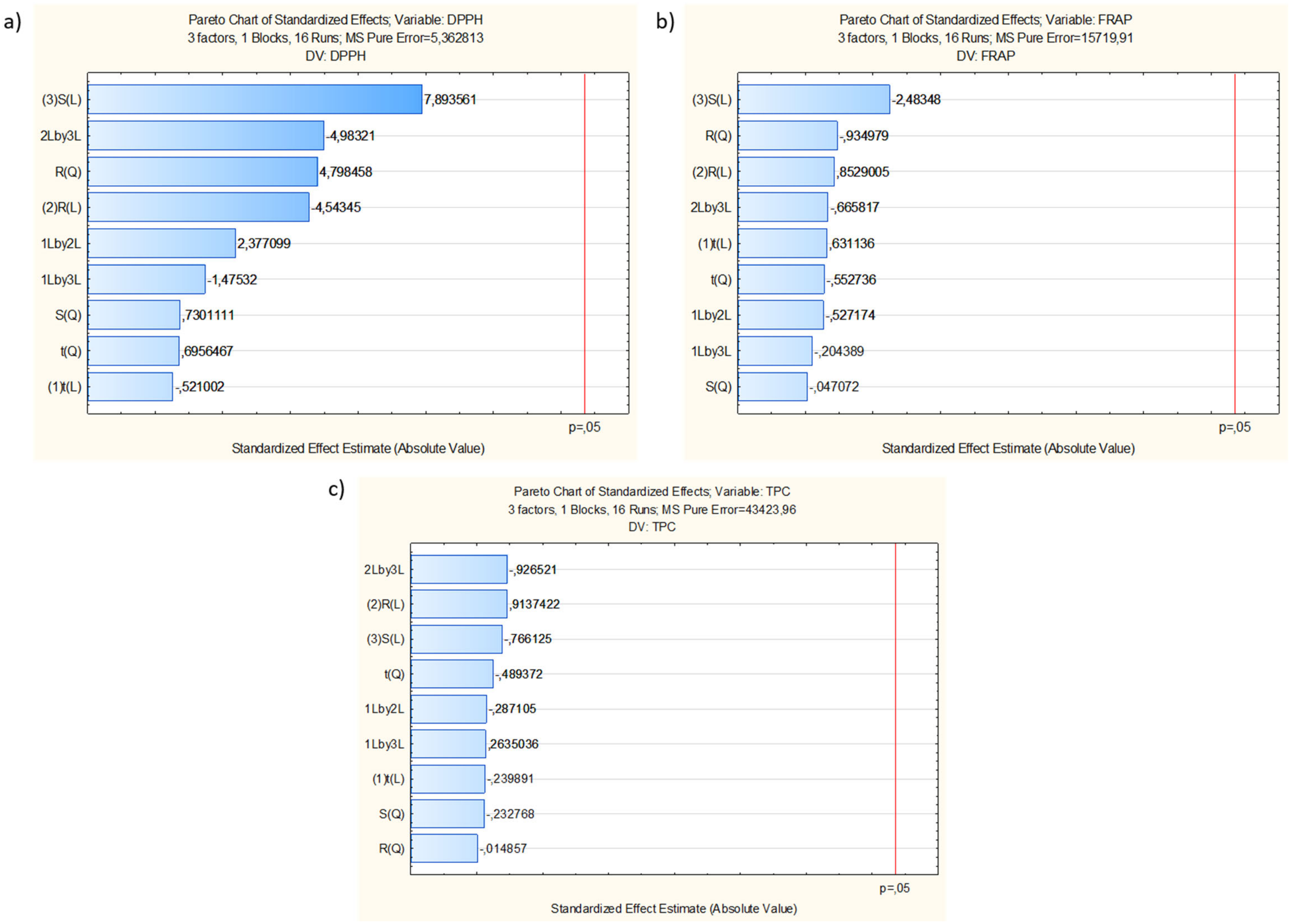

Figure S7. Pareto charts of the standardized effects of independent variables t, R and S in dependent variables DPPH (a), FRAP (b) and TPC (c) in the ultrasonic probe-assisted extraction.

Table S17. Regression coefficients, standard error (SE) and *p*-value of the second-order polynomial models for optimization of the extraction of antioxidants from *F. spiralis*, using ultrasonic probe-assisted extraction. t: extraction time (s); R: biomass:solvent ratio (g biomass/100 mL solvent); S: solvent (% of water in water:ethanol mixture); L: Linear effect; Q: Quadratic effect; 1Lby2L: interaction between the linear effect of t and the linear effect of R; 1Lby3L: interaction between the linear effect of t and the linear effect of S; 2Lby3L: interaction between the linear effect of R and the linear effect of S. Significant parameters are presented in red.

	DPPH						*FRAP*						*TPC*					
	Coeff.	SE	t(1)	p	-95,%	+95,%	Coeff.	SE	t(1)	p	-95,%	+95,%	Coeff.	SE	t(1)	p	-95,%	+95,%
Interc.	10,65	7,08	1,50	0,374	-79,33	100,62	-18,96	383,39	-0,05	0,969	-4890,40	4852,49	-92,25	637,21	-0,14	0,908	-8188,75	8004,25
(1)t (L)	-0,05	0,05	-1,06	0,481	-0,65	0,55	2,15	2,55	0,84	0,554	-30,25	34,56	1,62	4,24	0,38	0,767	-52,24	55,48
t (Q)	0,00	0,00	0,70	0,613	0,00	0,00	0,00	0,01	-0,55	0,679	-0,08	0,08	-0,01	0,01	-0,49	0,710	-0,14	0,13
(2)R (L)	-0,93	0,25	-3,71	0,167	-4,12	2,26	18,71	13,58	1,38	0,400	-153,81	191,23	14,96	22,57	0,66	0,627	-271,77	301,69
R (Q)	0,02	0,00	4,80	0,131	-0,03	0,07	-0,19	0,20	-0,93	0,521	-2,76	2,38	-0,01	0,34	-0,01	0,991	-4,28	4,27
(3)S (L)	0,44	0,12	3,75	0,166	-1,04	1,92	-0,18	6,31	-0,03	0,982	-80,33	79,98	3,19	10,48	0,30	0,812	-130,03	136,41
S (Q)	0,00	0,00	0,73	0,599	-0,01	0,01	0,00	0,05	-0,05	0,970	-0,59	0,59	-0,02	0,08	-0,23	0,854	-1,00	0,97
1L by 2L	0,00	0,00	2,38	0,254	-0,01	0,01	-0,02	0,04	-0,53	0,691	-0,51	0,47	-0,02	0,06	-0,29	0,822	-0,84	0,80
1L by 3L	0,00	0,00	-1,48	0,379	0,00	0,00	0,00	0,02	-0,20	0,872	-0,24	0,23	0,01	0,03	0,26	0,836	-0,38	0,40
2L by 3L	-0,01	0,00	-4,98	0,126	-0,03	0,01	-0,07	0,10	-0,67	0,626	-1,40	1,26	-0,16	0,17	-0,93	0,524	-2,37	2,05

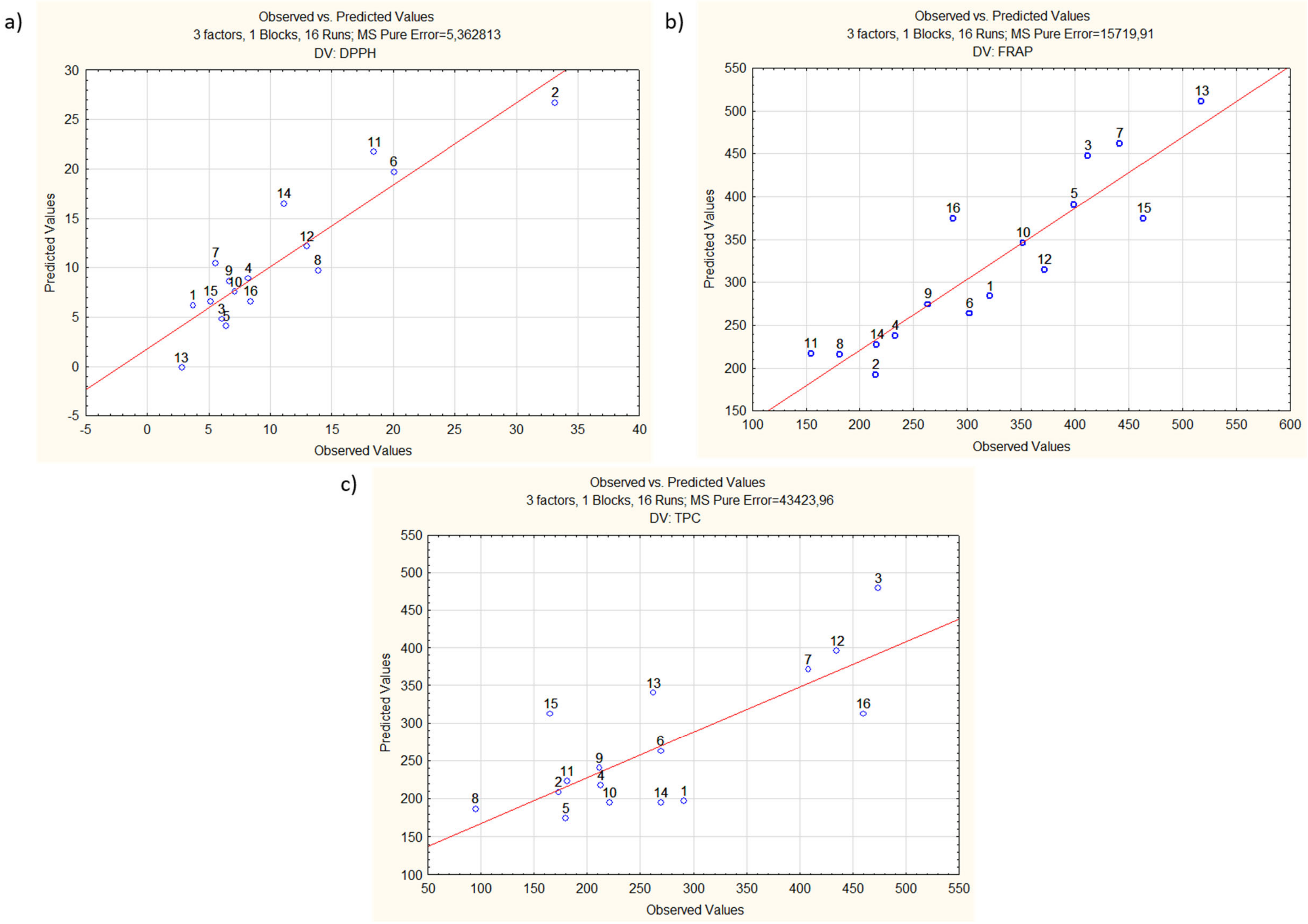

Figure S8. Observed vs. predicted DPPH (a), FRAP (b) and TPC (c) values for the ultrasonic probe-assisted extraction.

Table S18. Observed, predicted, and residual values for the ultrasonic probe-assisted extraction.

	DPPH			*FRAP*			*TPC*		
	Observed	Predictd	Resids	Observed	Predictd	Resids	Observed	Predictd	Resids
1	3,73	6,19	-2,46	321,27	283,62	37,65	291,17	197,19	93,98
2	33,18	26,66	6,52	215,36	192,26	23,10	173,29	208,48	-35,19
3	6,07	4,76	1,31	412,24	447,26	-35,02	474,16	479,06	-4,91
4	8,21	8,91	-0,70	233,05	237,84	-4,78	213,30	217,31	-4,01
5	6,43	4,06	2,37	399,73	391,31	8,42	179,60	173,61	5,99
6	20,06	19,70	0,36	302,36	263,70	38,66	269,45	262,56	6,89
7	5,57	10,42	-4,86	442,01	461,47	-19,46	408,05	370,88	37,17
8	13,86	9,74	4,13	181,79	215,80	-34,01	94,78	186,78	-92,00
9	6,65	8,62	-1,97	263,77	274,47	-10,70	211,72	240,43	-28,70
10	7,14	7,52	-0,38	352,05	346,49	5,55	220,83	194,93	25,90
11	18,46	21,69	-3,23	154,91	217,28	-62,36	181,25	222,91	-41,66
12	12,99	12,11	0,88	371,83	314,61	57,22	435,08	396,22	38,86
13	2,79	-0,18	2,97	517,85	511,10	6,75	262,35	340,02	-77,68
14	11,14	16,46	-5,32	215,79	227,69	-11,90	269,59	194,71	74,88
15	5,14	6,57	-1,44	463,98	374,88	89,10	165,33	312,44	-147,11
16	8,41	6,57	1,84	286,66	374,88	-88,21	460,03	312,44	147,59

2.5. High pressure

Table S19. Analysis of variance (ANOVA) of the second-order polynomial model for optimization of the extraction of antioxidants from *F. spiralis*, using high pressure-assisted extraction. t: extraction time (s); P: pressure (MPa); S: solvent (% of water in water:ethanol mixture); SS: Sum of squares; df: Degree of freedom; MS: Mean square; F: F value; p: probability; L: Linear effect; Q: Quadratic effect; 1Lby2L: interaction between the linear effect of t and the linear effect of P; 1Lby3L: interaction between the linear effect of t and the linear effect of S; 2Lby3L: interaction between the linear effect of P and the linear effect of S. Significant parameters are presented in red.

	DPPH					*FRAP*					*TPC*				
	SS	df	MS	F	p	SS	df	MS	F	p	SS	df	MS	F	p
(1)t (L)	7161,50	1	7161,54	606,26	<0,001	6885,40	1	6885,36	1,70	0,250	579,39	1	579,39	0,20	0,670
t (Q)	24121,30	1	24121,32	2041,98	0,000	34,70	1	34,70	0,01	0,930	3537,27	1	3537,27	1,25	0,314
(2)P (L)	1935,50	1	1935,49	163,85	<0,001	5189,30	1	5189,26	1,28	0,310	1,85	1	1,85	0,00	0,981
P (Q)	5297,80	1	5297,78	448,48	<0,001	31108,80	1	31108,85	7,66	0,039	32131,00	1	32131,00	11,36	0,020
(3)S (L)	17998,10	1	17998,10	1523,62	0,000	5312,50	1	5312,53	1,31	0,305	475,57	1	475,57	0,17	0,699
S (Q)	6827,70	1	6827,66	577,99	<0,001	5661,10	1	5661,13	1,39	0,291	4,06	1	4,06	0,00	0,971
1L by 2L	738,10	1	738,12	62,49	0,001	2459,60	1	2459,56	0,61	0,472	323,56	1	323,56	0,11	0,749
1L by 3L	5839,40	1	5839,36	494,33	<0,001	15860,60	1	15860,58	3,90	0,105	7187,60	1	7187,60	2,54	0,172
2L by 3L	2381,10	1	2381,09	201,57	<0,001	16245,60	1	16245,59	4,00	0,102	8688,22	1	8688,22	3,07	0,140
Lack of Fit	62276,40	5	12455,28	1054,39	0,000	4800,90	5	960,17	0,24	0,930	9097,78	5	1819,56	0,64	0,680
Pure Error	59,10	5	11,81			20310,40	5	4062,07			14142,91	5	2828,58		
Total SS	129790,30	19				111839,60	19				78795,30	19			
R^2			0,520					0.775					0.705		
R^2 *adj.*			0.087					0.573					0.440		

Table S20. Effect estimates of variables in high pressure-assisted extraction. t: extraction time (s); P: pressure (MPa); S: solvent (% of water in water:ethanol mixture); L: Linear effect; Q: Quadratic effect; 1Lby2L: interaction between the linear effect of t and the linear effect of P; 1Lby3L: interaction between the linear effect of t and the linear effect of S; 2Lby3L: interaction between the linear effect of P and the linear effect of S. Significant parameters are presented in red.

	Parameters	Effect	Std.Err. - Pure Err	t(1)	p	-95,% - Cnf.Limt	+95,% - Cnf.Limt	Coeff.	Std.Err. - Coeff.	-95,% - Cnf.Limt	+95,% - Cnf.Limt
DPPH	Mean/Interc.	3,67	1,40	2,62	0,047	0,07	7,28	3,67	1,40	0,07	7,28
	(1)t (L)	-45,82	1,86	-24,62	<0,001	-50,60	-41,04	-22,91	0,93	-25,30	-20,52
	t (Q)	81,95	1,81	45,19	<0,001	77,29	86,62	40,98	0,91	38,65	43,31
	(2)P (L)	-23,82	1,86	-12,80	<0,001	-28,60	-19,04	-11,91	0,93	-14,30	-9,52
	P (Q)	38,41	1,81	21,18	<0,001	33,75	43,07	19,20	0,91	16,87	21,53
	(3)S (L)	-72,64	1,86	-39,03	<0,001	-77,42	-67,85	-36,32	0,93	-38,71	-33,93
	S (Q)	43,60	1,81	24,04	<0,001	38,94	48,26	21,80	0,91	19,47	24,13
	1L by 2L	-19,21	2,43	-7,90	0,001	-25,46	-12,96	-9,61	1,22	-12,73	-6,48
	1L by 3L	54,03	2,43	22,23	<0,001	47,79	60,28	27,02	1,22	23,89	30,14
	2L by 3L	-34,50	2,43	-14,20	<0,001	-40,75	-28,26	-17,25	1,22	-20,38	-14,13
FRAP	Mean/Interc.	225,71	25,99	8,68	0,000	158,90	292,53	225,71	25,99	158,90	292,53
	(1)t (L)	-44,93	34,51	-1,30	0,250	-133,63	43,78	-22,46	17,25	-66,82	21,89
	t (Q)	3,11	33,63	0,09	0,930	-83,34	89,56	1,55	16,82	-41,67	44,78
	(2)P (L)	-39,00	34,51	-1,13	0,310	-127,71	49,70	-19,50	17,25	-63,85	24,85
	P (Q)	93,07	33,63	2,77	0,039	6,62	179,52	46,54	16,82	3,31	89,76
	(3)S (L)	39,46	34,51	1,14	0,305	-49,24	128,17	19,73	17,25	-24,62	64,08
	S (Q)	39,70	33,63	1,18	0,291	-46,75	126,16	19,85	16,82	-23,37	63,08
	1L by 2L	35,07	45,07	0,78	0,472	-80,78	150,92	17,53	22,53	-40,39	75,46
	1L by 3L	-89,05	45,07	-1,98	0,105	-204,90	26,80	-44,53	22,53	-102,45	13,40
	2L by 3L	90,13	45,07	2,00	0,102	-25,72	205,97	45,06	22,53	-12,86	102,99
TPC	Mean/Interc.	156,77	21,69	7,23	0,001	101,01	212,52	156,77	21,69	101,01	212,52
	(1)t (L)	-13,03	28,80	-0,45	0,670	-87,05	60,99	-6,52	14,40	-43,53	30,49
	t (Q)	-31,38	28,06	-1,12	0,314	-103,53	40,76	-15,69	14,03	-51,76	20,38
	(2)P (L)	-0,74	28,80	-0,03	0,981	-74,76	73,29	-0,37	14,40	-37,38	36,64
	P (Q)	94,59	28,06	3,37	0,020	22,45	166,73	47,29	14,03	11,22	83,36
	(3)S (L)	11,81	28,80	0,41	0,699	-62,21	85,83	5,90	14,40	-31,11	42,91
	S (Q)	-1,06	28,06	-0,04	0,971	-73,20	71,08	-0,53	14,03	-36,60	35,54
	1L by 2L	12,72	37,61	0,34	0,749	-83,95	109,39	6,36	18,80	-41,98	54,70
	1L by 3L	-59,95	37,61	-1,59	0,172	-156,62	36,72	-29,97	18,80	-78,31	18,36
	2L by 3L	65,91	37,61	1,75	0,140	-30,76	162,58	32,95	18,80	-15,38	81,29

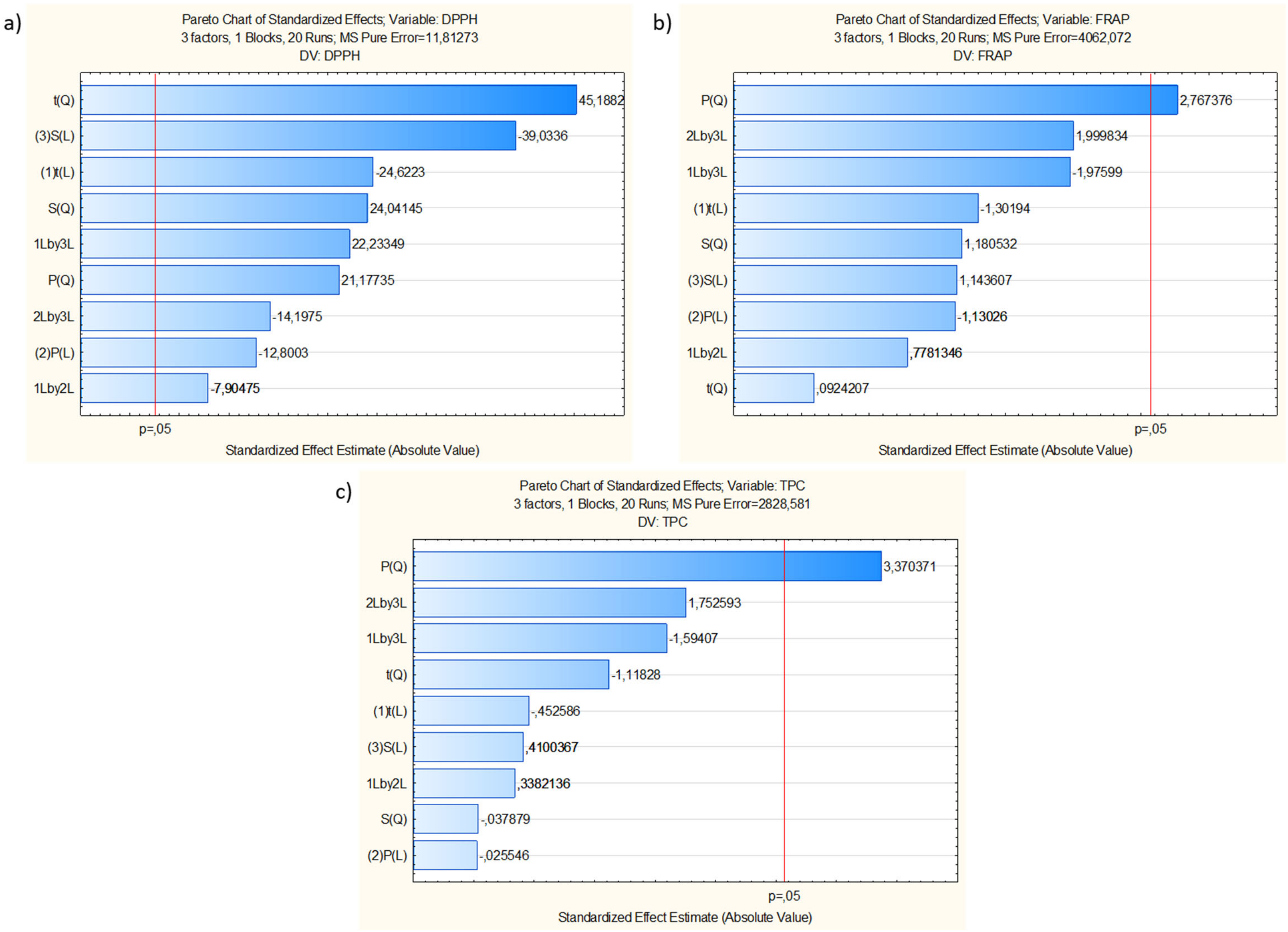

Figure S9. Pareto charts of the standardized effects of independent variables t, R and S in dependent variables DPPH (a), FRAP (b) and TPC (c) in the high pressure-assisted extraction.

Table S21. Regression coefficients, standard error (SE) and *p*-value of the second-order polynomial models for optimization of the extraction of antioxidants from *F. spiralis*, using high pressure-assisted extraction. t: extraction time (s); P: pressure (MPa); S: solvent (% of water in water:ethanol mixture); L: Linear effect; Q: Quadratic effect; 1Lby2L: interaction between the linear effect of t and the linear effect of P; 1Lby3L: interaction between the linear effect of t and the linear effect of S; 2Lby3L: interaction between the linear effect of P and the linear effect of S. Significant parameters are presented in red.

	DPPH						*FRAP*						*TPC*					
	Coeff.	SE	t(1)	p	-95,%	+95,%	Coeff.	SE	t(1)	p	-95,%	+95,%	Coeff.	SE	t(1)	p	-95,%	+95,%
Interc.	501,12	9,96	50,33	<0,001	475,52	526,71	494,82	184,64	2,68	0,044	20,19	969,46	207,80	154,08	1,35	0,235	-188,27	603,86
(1)t (L)	-0,55	0,01	-46,79	<0,001	-0,58	-0,52	0,03	0,22	0,16	0,879	-0,52	0,59	0,24	0,18	1,32	0,244	-0,23	0,71
t (Q)	0,00	0,00	45,19	<0,001	0,00	0,00	0,00	0,00	0,09	0,930	0,00	0,00	0,00	0,00	-1,12	0,314	0,00	0,00
(2)P (L)	-0,14	0,03	-5,25	0,003	-0,21	-0,07	-1,64	0,49	-3,33	0,021	-2,91	-0,37	-1,29	0,41	-3,13	0,026	-2,34	-0,23
P (Q)	0,00	0,00	21,18	<0,001	0,00	0,00	0,00	0,00	2,77	0,039	0,00	0,00	0,00	0,00	3,37	0,020	0,00	0,00
(3)S (L)	-4,84	0,16	-30,39	<0,001	-5,25	-4,43	-0,60	2,95	-0,20	0,846	-8,20	6,99	0,77	2,47	0,31	0,768	-5,57	7,10
S (Q)	0,02	0,00	24,04	<0,001	0,02	0,03	0,02	0,02	1,18	0,291	-0,03	0,07	0,00	0,02	-0,04	0,971	-0,04	0,04
1L by 2L	0,00	0,00	-7,90	0,001	0,00	0,00	0,00	0,00	0,78	0,472	0,00	0,00	0,00	0,00	0,34	0,749	0,00	0,00
1L by 3L	0,00	0,00	22,23	<0,001	0,00	0,00	0,00	0,00	-1,98	0,105	-0,01	0,00	0,00	0,00	-1,59	0,172	-0,01	0,00
2L by 3L	0,00	0,00	-14,20	<0,001	0,00	0,00	0,01	0,00	2,00	0,102	0,00	0,02	0,01	0,00	1,75	0,140	0,00	0,02

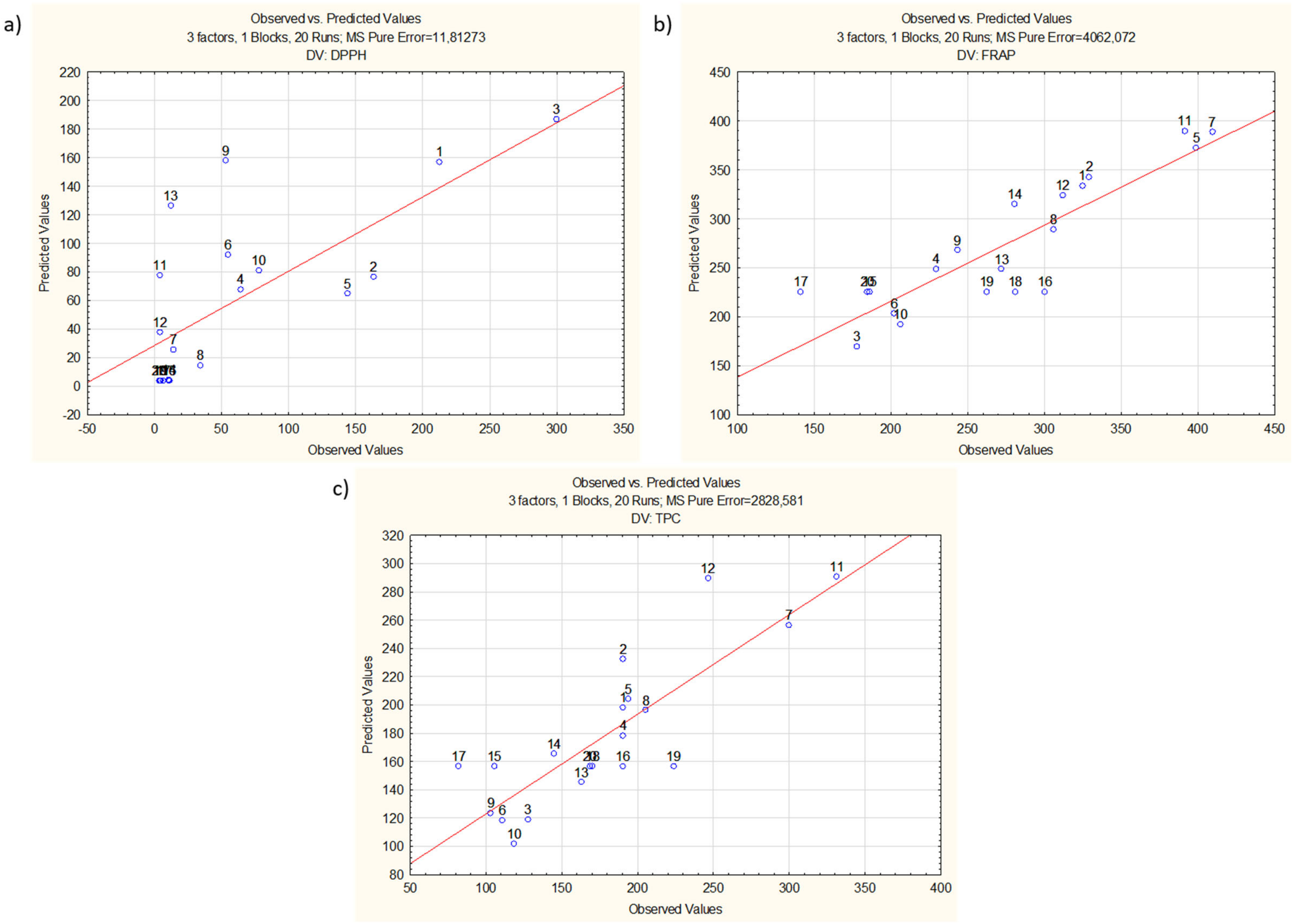

Figure S10. Observed vs. predicted DPPH (a), FRAP (b) and TPC (c) values for the high pressure-assisted extraction.

Table S22. Observed, predicted, and residual values for the high pressure-assisted extraction.

	DPPH			*FRAP*			*TPC*		
	Observed	Predictd	Resids	Observed	Predictd	Resids	Observed	Predictd	Resids
1	212,83	156,95	55,88	324,92	333,96	-9,04	190,66	198,16	-7,50
2	163,45	76,31	87,14	329,35	343,01	-13,67	190,62	232,35	-41,74
3	300,00	186,85	113,15	178,07	169,76	8,31	127,89	118,79	9,10
4	64,31	67,78	-3,47	229,72	248,95	-19,24	190,42	178,43	11,99
5	143,97	64,79	79,18	399,01	372,35	26,67	193,86	204,00	-10,14
6	54,77	92,21	-37,44	202,42	203,30	-0,88	111,05	118,30	-7,25
7	14,24	25,67	-11,43	409,50	388,40	21,10	300,04	256,46	43,59
8	34,50	14,68	19,83	305,96	289,49	16,47	205,55	196,20	9,35
9	53,36	157,82	-104,46	243,42	267,84	-24,42	103,45	123,42	-19,97
10	78,01	80,84	-2,83	206,25	192,36	13,89	118,88	101,53	17,35
11	4,39	77,88	-73,49	391,56	389,82	1,75	331,42	290,86	40,55
12	4,06	37,87	-33,80	312,01	324,29	-12,28	246,45	289,63	-43,17
13	12,28	126,22	-113,94	272,19	248,59	23,60	162,99	145,35	17,64
14	10,83	4,19	6,64	280,76	314,89	-34,13	144,92	165,18	-20,26
15	11,11	3,67	7,44	186,15	225,71	-39,57	105,59	156,77	-51,18
16	11,09	3,67	7,42	300,31	225,71	74,60	190,37	156,77	33,61
17	6,46	3,67	2,79	141,46	225,71	-84,25	82,16	156,77	-74,60
18	4,06	3,67	0,38	281,04	225,71	55,33	170,54	156,77	13,78
19	4,80	3,67	1,13	262,40	225,71	36,68	223,85	156,77	67,08
20	3,57	3,67	-0,10	184,79	225,71	-40,92	168,55	156,77	11,78
